# Metal (Mo, W, Ti) Carbide Catalysts: Synthesis and Application as Alternative Catalysts for Dry Reforming of Hydrocarbons—A Review

**DOI:** 10.3390/ijms222212337

**Published:** 2021-11-15

**Authors:** Natalia Czaplicka, Andrzej Rogala, Izabela Wysocka

**Affiliations:** Department of Process Engineering and Chemical Technology, Faculty of Chemistry, Gdansk University of Technology, Narutowicza 11/12 St., 80-233 Gdansk, Poland; natalia.czaplicka@pg.edu.pl (N.C.); andrzej.rogala@pg.edu.pl (A.R.)

**Keywords:** dry reforming, catalysts, metal carbides, molybdenum, tungsten, titanium

## Abstract

Dry reforming of hydrocarbons (DRH) is a pro-environmental method for syngas production. It owes its pro-environmental character to the use of carbon dioxide, which is one of the main greenhouse gases. Currently used nickel catalysts on oxide supports suffer from rapid deactivation due to sintering of active metal particles or the deposition of carbon deposits blocking the flow of gases through the reaction tube. In this view, new alternative catalysts are highly sought after. Transition metal carbides (TMCs) can potentially replace traditional nickel catalysts due to their stability and activity in DR processes. The catalytic activity of carbides results from the synthesis-dependent structural properties of carbides. In this respect, this review presents the most important methods of titanium, molybdenum, and tungsten carbide synthesis and the influence of their properties on activity in catalyzing the reaction of methane with carbon dioxide.

## 1. Introduction

Synthetic gas, called syngas, is one of the most important intermediates for the production of fuels, acetic acid, ammonia, and methanol, and the Fischer–Tropsch process. It is a mixture of carbon monoxide and hydrogen, and very often carbon dioxide. Among the technologies for syngas production, steam reforming, dry reforming (DR), partial oxidation (PO), and autothermal reforming (ATR) can be distinguished [1]. Among the mentioned technologies, steam and dry reforming are highly endothermic processes. Their standard enthalpies are +206 kJ/mol and +247 kJ/mol, respectively. In contrast to DR and ST, the enthalpies of ATR and PO are negative as a result of the ongoing oxidation reaction. Each of the processes is characterized by other process parameters and the hydrogen-to-carbon monoxide ratio in the outlet stream (see Table 1). Dry reforming of hydrocarbons (DRH) is a pro-environmental technique for synthetic gas generation. DR processes involve the processing of hydrocarbons, mainly methane, and carbon dioxide, which are the most important greenhouse gases. Furthermore, dry reforming is a suitable method to manage biogas emitted during biomass fermentation or digestion of anaerobic microorganisms [2]. The hydrogen-to-carbon monoxide ratio in the outlet stream is around 1.0. A ratio of n = 1 is favorable in many processes, such as ammonia, methanol, dimethyl ether, and selective Fisher–Tropsch synthesis. Furthermore, DR progresses under atmospheric pressure, allowing the use of an apparatus that does not have to withstand high pressures [3,4,5,6].

Processes of dry reforming, mainly dry reforming of methane (DRM), are still industrially immature processes due to the fast catalyst deactivation. Currently, the most widely used catalysts are based on nickel particles supported on metal oxides such as aluminum oxide, magnesium oxide, silicon oxide, zirconium oxide, lanthanum oxide, and magnesium-aluminum spinel with alkali metal promoters [3,7,8]. The use of nickel catalysts is economically justified because of their relatively low price and high activity, comparable to the activity of noble metals. The endothermic character of the DRM reaction (+247 kJ/mol) requires a large amount of heat to be provided, which causes sintering and growth of agglomerates of active phase particles, leading to a reduction in the specific surface area, a reduction in the number of active sites, and increased deposition of carbon structures including coke and unsaturated polyaromatic hydrocarbons with H/C ratios less than unity [7]. With regard to the above factors, current worldwide research in the field is focused on the development of new catalysts with higher activity and stability in the dry reforming process [8].

Transition metal carbides (TMCs) have attracted much interest because of their high thermal stability, good electronic properties, and catalytic activity. TMCs are a wide group of catalysts currently developed for many catalytic processes, such as hydrocarbon reforming, hydrogenation, and CO oxidation. Under DRM conditions, they participate in recarburization–oxidation cycles. In the oxidation reaction, carbon dioxide is reduced into carbon monoxide. On the other hand, during the recarburization reaction, carbon atoms from methane cracking and carbon monoxide disproportionation are built into the carbide structure, thus preventing the formation of carbon deposits on the surface of the catalyst [9,10]. 

Dissociative adsorption and activation of methane and carbon dioxide, as well as carbon formation steps, are sensitive to the structure, geometric, and electronic properties of catalysts. The catalytic activity of catalysts is strictly dependent on the morphological properties of the active phase and the support, including the pore structure, size, shape, and distribution of the active phase, the support, and the modifiers [11,12]. The structure of TMCs is developed through the incorporation of carbon atoms into interstitial sites of transition metals. The method of preparation strongly affects the morphology of the carbides and therefore their catalytic properties.

**Figure 1:**
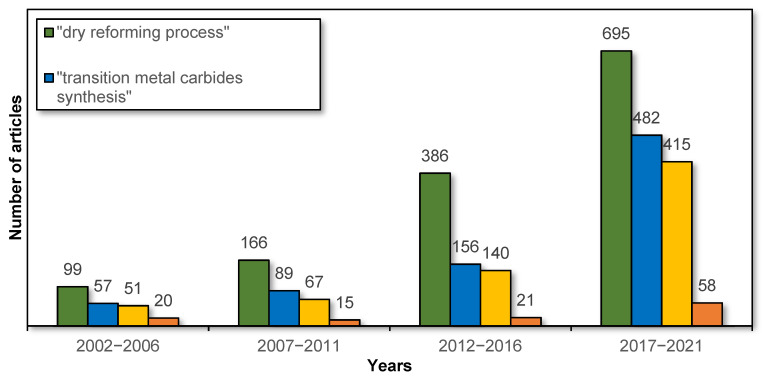
Number of articles in particular years regarding the dry reforming process and TMCs depending on the phrase entered (data from the Scopus database).

Over the past 20 years, there has been a significant increase in interest in both the dry reforming process and transition metal carbide catalysts, including their synthesis and applications in the DR process. This is confirmed by the data from the Scopus database on the number of articles on this subject in particular years, as shown in Figure 1. However, there are still few reviews of this topic in the literature. In this regard, the aim of this study is to review recent developments in the fields of preparation methods and their effect on the activity of transition metal (molybdenum, tungsten, and titanium) carbides in the dry reforming of hydrocarbons. 

## 2. Metal Carbides 

Carbide compounds may be divided into three groups: salinic, intermediate, and interstitial. The first two groups are considered to be unstable even at low temperatures and easily degraded by water. The interstitial compounds are characterized by unique properties due to special bonding between carbon and transition metal atoms. The chemical bonds in transition metal carbides are quite complex. Covalent, metallic, and ionic bonding may be distinguished. The first type of bonding refers to metal–carbon bonding, metallic bonding refers to metal–metal interactions, and ionic bonding is attributed to charge transfer between metals and carbon. This is reflected in their properties such as high hardness and high melting points, which are typical properties for solids with ionic or covalent bonding, while thermal and electrical conductivity is characterized by metallic bonds. The electronic structure of transition metal carbides has been widely studied in the literature, where it has been shown that the main feature of the chemical bond in transition metal carbides is the covalent bond with the *2p* hybridization of carbon atoms and the *d* orbital of the metal atom. Transition metal carbides have found applications in industry. They are used as additives to materials for improving their strength [13], cermet materials [14], protective coatings [15], refining layers of cutting tool blades [16,17], and balls and ball bearing raceways [18]. Metal carbides have also found application in various branches of catalysis, such as hydrotreatment including hydrodesulfurization, hydrodenitrogenation, and hydrodeoxygenation—for removal of heteroatoms (S, N, O) from hydrocarbons. The most widely examined carbide is molybdenum carbide with cobalt or nickel carbides [19,20]; however, carbides WC, NbC, VC, and TiC have also been examined [19,21]. Other examples of application of carbides in catalysts are hydrogenolysis and isomerization of hydrocarbons [22], catalytic and electrocatalytic hydrogen generation [23,24,25], hydrocarbon reforming [26,27], carbon dioxide upgrading [23,28], hydrogenation [29], and aromatization [30]. Moreover, carbides of layered structures are receiving increased attention in gas sensing [31,32] and battery applications [27,33,34].

### 2.1. Tungsten Carbide

Tungsten carbide contains two separate crystalline phases, W_2_C and WC, which, due to a different structure, are characterized by different ranges of temperature stability [35]. The β-W_2_C phase is stable at lower temperatures compared to α-WC and contains several structural modifications due to the different arrangement of carbon atoms [35]. Meanwhile, the α-WC phase has a hexagonal structure in which the carbon atoms are in the center of the tungsten trigonal body. Thus, in its most basic form, the crystals of WC have a hexagonal structure, and it is a fine gray powder. It is characterized by high strength, fracture resistance, and resistance to high temperature and abrasion, as well as high melting (2600–2850 °C) and boiling points (6000 °C) [36]. Due to its properties, WC is widely used, among others, in the chemical, armament, and electronics industries, in the production of cutting mechanical tools, and in abrasives and surface coatings [33]. In addition, tungsten carbide exhibits catalytic properties, and the efficiency of WC as a catalyst is similar to that of platinum [34], its use being associated with much higher costs. Therefore, the subject of many studies is the use of WC in chemical and electrochemical catalysis, which allows one to reduce the cost of the process by partially or completely replacing noble metals with tungsten carbide. The literature includes studies on the use of WC as a highly active catalyst for the isomerization of alkanes [34], for the decomposition of hydrazine [37], for ethylene hydrogenation [38], for methane reforming [39], for the conversion of cellulose to ethylene glycol [40], for methanol decomposition [41], and in many other processes. The synergistic effect of WC with platinum or palladium in electrocatalysis has also been demonstrated. This means that tungsten carbide as an electrocatalyst promoter can improve the electrocatalytic effect and partially replace noble metals [42,43]. The advantages of WC as a catalyst also include high resistance to acid solutions and resistance to CO poisoning, resulting in an extended catalytic life [34].

### 2.2. Molybdenum Carbide

Molybdenum carbide is characterized by high thermal stability, good thermal and electrical conductivity, resistance to corrosion, hardness, and a melting point above 2000 °C. Due to their properties, molybdenum carbides have found applications in catalysis, electrocatalysis, anti-creeping alloys, and as cutting tool parts. Three basic forms: MoC, Mo_2_C, and MoC_1-x_, of molybdenum carbide can be distinguished. The metal in the lattice may form hexagonal (hex), hexagonal close-packed (hcp), and face-centered cubic (fcc) structures. Mo_2_C carbide may exhibit cubic (y, hexagonal (α), and orthorhombic (β) phases [11,44,45]. Molybdenum carbide has already found application in the synthesis and decomposition of ammonia, hydrocarbons, oxidation, hydrogen generation, hydrogenation, photocatalytic oxidation, water splitting, hydrodesulfurization, methane aromatization, and hydrocarbon reforming. The catalytic properties of molybdenum carbide are very comparable to the activity of noble metals [46,47,48,49]. 

### 2.3. Titanium Carbide

Titanium carbide crystals have a face-centered cubic structure with a lattice constant of a = 4.328 Å [44]. Titanium carbide is classified as an interstitial metal carbide because it is very often a nonstoichiometric compound (TiC_x_). Homogeneous samples are obtained in the range of x values from 0.5 to 0.98, where some of the positions of carbon atoms are vacant [45,50]. TiC, similar to WC, is one of the high-melting compounds, which is widely applied in many industries as a component of carbidosteels, hard-alloy and cutting tools, and abrasive materials, as well as in the manufacture of ceramics and alloys [51]. This is due to its characteristic properties such as its high melting point (3160 °C), hardness, heat resistance, elasticity modulus, wear and crack resistance, and fatigue limit [51,52]. Moreover, many applications of TiC as a catalyst can be found in the literature. Titanium carbide-supported catalysts are used in CO_2_ hydrogenation and methanol synthesis [53,54], electrochemical reduction of CO_2_ to CH_4_ [55], oxygen reduction reactions [56,57], water–gas shift reactions [58], CO oxidation [59], and many others. Generally, the use of TiC as a support has been shown to be effective in improving the stability of Pt-based electrocatalysts [60]. It has been reported that titanium carbide can also be used as a catalyst in dry reforming. 

## 3. Synthesis of Metal Carbides

### 3.1. Reactive Sintering and Temperature-Programmed Reduction (TPR) 

Temperature-programmed reduction (TPR) or carburization (TPC) and reactive sintering are the most commonly used methods for metal carbide preparation. In general, the TPR/TPC preparation method involves three steps: (1) preparation of metal (in the form of salts or oxides) and carbon sources, (2) carbothermal reduction at high temperatures under a reductive atmosphere and solid-state reaction, and (3) stabilization through passivation.

In the case of the traditional industrial method, the WC powder is commercially synthesized by carbonization of W together with C at 1400–1600 °C in an atmosphere of flowing hydrogen for 2–10 h. Tungsten powder is first produced using very pure tungsten trioxide, tungsten acid (hydrated trioxide), or ammonium paratungstate (APT), and then carbonized to WC [61]. There are many studies in the literature in which powder WC was obtained by many different methods including mechanical melting [62], thermochemical reaction [63], thermal decomposition of metal complexes [64], chemical vapor deposition [65], combustion synthesis [66], or solid-state metathesis [67]. However, these common methods for preparing WC nanopowders face enormous challenges such as high cost, low yield, contamination of the final product, and a wide particle size distribution [68]. Modified WC may also be prepared using the TPR method. This method involves the preparation of tungsten precursors, calcination, reduction of the methane stream in hydrogen, and passivation [69]. 

The synthesis of molybdenum carbide by temperature-programmed reduction involves the preparation of the molybdenum precursor (e.g., MoO_3_, MoO_2,_ NiMoO_4_) and further annealing under hydrogen and carbon-containing gases [70,71,72,73]. In most reports, methane is used as a carbon-containing gas; however, other mixtures have been examined including ethane, propane, and butane in hydrogen [9,74,75,76]. Yao et al. [69] synthesized Mo_2_C modified with nickel using the following steps: preparation of the Ni-Mo oxide precursor from an aqueous solution of metal salts, calcination of the obtained precursor, and TPR under 40% CH_4_ in H_2_ flow at a temperature in the range of 300–800 °C. The final step was passivation in 1%O_2_/Ar for 12 h. The selection of the carbonaceous gas and its concentration determine the final crystal structure and surface properties of the carbides. A carbon source may also be in solid form, and it acts as a reducing agent and support. Biochar [77], biomass [24], resins [78], and carbon nanotubes [79] have been investigated. Liang et al. [78] investigated the effect of the preparation procedure on the formation of α-Mo_1-x_C and β-Mo_2_C, and the solid mixture of α-Mo_1-x_C and β-Mo_2_C. Molybdenum carbide was prepared by ion exchange, impregnation, and mechanical mixing of molybdate salt with a strong alkali anion exchange resin and heated under a hydrogen or argon atmosphere in a temperature range of 350 to 900 °C. They found that annealing under H_2_ promotes the formation of the beta phase. The two identified possible phase formation mechanisms were topotactic and nontopotactic transition. In the case of nontopotactic formation, the mechanism proceeds through the formation of β-Mo_2_C from MoO_2_ or MoO_x_, while for the topotactic route, the formation of α-Mo_1-x_C from MoO_x_ through MoO_x_C_y_ is observed. Moreover, the formation of particular crystal phases depends on the temperature. Below 500 °C carburization, no XRD reflections or very weak ones are observed for possible crystal molybdenum carbide phases. A higher temperature is required—above 700 °C for the formation of Mo_2_C, MoC_1-x_, or MoO_x_C_y_. The TPR/TPC method is the most commonly used method for the preparation of supported molybdenum carbide. In the first step, molybdenum salt is dissolved in water or in an aqueous solution of a stabilizer (e.g., citric acid). The support (Al_2_O_3_, SiO_2_, SiC) is then immersed in the molybdenum salt solution. After the adsorption step, the impregnated solid is calcined under air atmosphere and carburized [10,73]. The TPR/TPC method is associated with impurities of polymeric carbon from carbon-carried gas pyrolysis. The contaminated surface may be purified from polymeric carbon by treatment with a hydrogen stream [70,78]. 

TiC is a commercially available material and, in this form, has been used by scientists in various research works [80,81,82]. Its advantage is definitely its morphology, defined by the manufacturer in advance, which, however, is associated with significant purchase costs. Due to the high cost of commercial TiC, new alternative methods of synthesizing this compound are sought, which could reduce its cost. The literature is dominated by high-temperature methods of obtaining titanium carbide. In the three-step method for obtaining TiC proposed by Xie et al. [83], the first step involved the preparation of the titanium precursor via the sol–gel method from tetrabutyl titanate in acidic media. The carbon source (phenol-formaldehyde resin) was added to the alcoholic solution of an organometallic salt. After the hydrolysis, the precipitate was aged, dried, milled, and subjected to vacuum sintering. The final step involved high-temperature purification under a hydrogen or hydrogen/argon atmosphere. 

Other methods of TiC production, which require a high energy input, are carbothermal reduction of TiO_2_ [84,85,86], the plasma method [87], synthesis from elements [88], the self-propagating high-temperature method [89], chemical vapor deposition [90], and the magnesiothermic method [91,92]. The simplest, relatively inexpensive, most common, and most commercially used method is carbothermal reduction. However, due to the kinetic barrier, this process requires a high temperature of about 1700–2300 °C, which results in a high energy consumption and high cost. Furthermore, the synthesis of TiC using this method is characterized by low yield [84,86]. In the case of synthesis from elements, the main limiting factor is the high cost of titanium powder. However, this method is highly exothermic and therefore requires a low temperature. Additionally, synthesis from elements is characterized by high efficiency [88]. Therefore, as with tungsten carbide, the ball milling method, the precursor method, and molten salt synthesis are used to reduce the temperatures needed to synthesize TiC.

### 3.2. High-Energy Mechanical ball Milling Technique

One of the most common methods for producing carbide nanopowders is the high-energy mechanical ball milling technique, which allows reducing the size of powder particles with micron or submicron sizes [93,94,95,96]. Moreover, the mechanical activation of substrates allows for higher conversion efficiencies. Various milling techniques are used including planetary mills and abrasive mills [97,98,99]. Although mechanical milling techniques are versatile in creating nanostructured materials, most conventional mechanical milling techniques have the disadvantages of long processing times (usually more than 15 h), contamination, high energy inputs, and a relatively small volume of the powder obtained, due to the approximately ten times smaller proportion of substrates than the grinding balls in the total surface of the mill. However, these disadvantages can be minimized by optimizing process variables [93,94]. In addition, to prevent the oxidation of metals to metal oxides, the entire milling procedure is performed under an inert gas atmosphere. The mechanical milling process can also be used to induce a chemical reaction during milling, which is then termed mechanochemical synthesis. Table 2, Table 3 and Table 4 contain the selected process parameters for the synthesis of WC, TiC, and Mo_2_C, respectively, by mechanical milling and the characteristics of the obtained particles. The main inert gas used with this method is argon. As starting materials, metal powders (Mo, W, and Ti) or metal oxides (MoO_3_, WO_3_) are commonly used, with graphite as a carbon source. In the case of titanium carbide, mainly due to the high stability of titanium dioxide, it is hardly used. Recently, Sheybani and Javadpour [100] obtained Mo_2_C during the carbothermic reduction of molybdenite in the presence of sodium carbonate. They found that mechanical activation through milling for 70 h is crucial for the reaction leading to Mo_2_C formation. Furthermore, the presence of Na_2_CO_3_ significantly improved the yield of molybdenum carbide synthesis. They observed that after 70 h of mechanical activation from the MoS_2_, C, and Na_2_CO_3_ products, reductions of Na_2_MoO_4_ to Na_2_S and MoO_2_ to Mo_2_C occurred. High-energy mechanical ball milling may also be a preliminary step for further processing, for example, synthesis in arc plasma [101].

### 3.3. Structure-Directing Methods

Another method of synthesizing tungsten and titanium carbides is the precursor method. In the case of WC, the carbothermal reduction process involves the use of a carbon-coated WO_3_ precursor for the preparation of WC powders [107,108]. This allows for the production of WC by direct reduction of tungsten oxide with carbon. The process consists of two steps in which the oxide powders are first coated with carbon by cracking the gaseous hydrocarbon, propylene (C_3_H_6_), and then mixed with a substantial amount of carbon black and post-treated at temperatures ranging from 600 to 1400°C in a flowing argon atmosphere or H_2_-Ar for WC synthesis. The advantages of this process include increasing the contact surface between WO_3_ and carbon, as well as increasing the reaction rate, and thus reducing the reaction time [68]. However, this method uses carbon black, high-purity gases, and nanometric WO_3_ as reactants, which is not commercially profitable. Moreover, the particle size of the obtained WC powder is submicron [107]. Therefore, to overcome the disadvantages of the conventional method, other methods of synthesizing nanostructured WC powders from various precursors are sought. However, the hydrothermal reaction is an effective method of obtaining the core–carbon shell structure, which is becoming increasingly popular and does not require an expensive reducing gas or complicated equipment [109]. Metal salts, e.g., ammonium metatungstate (AMT) or ammonium paratungstate (APT), and organic compounds, e.g., glycine, corn starch, chitin, and iota-carrageenan, are used as input materials in this process. In the case of using such reagents, first, water and ammonium ions evaporate, resulting in the formation of WO_3_. Then, carbon from organic sources reduces WO_3_, which provides WC. This method can be used for the synthesis of carbon-coated tungsten oxide structures that could be directly used to produce WC nanopowders by in situ reduction and carburization. Selected preparation parameters for WC synthesis by the precursor method and the characteristics of the particles obtained are summarized in Table 5.

Structure-directing synthesis of molybdenum carbide is based mainly on the MoO_x_-amine hybrid precursor. The use of amines in the synthesis of molybdenum carbides allows the preparation of catalysts with 1D, 2D, or 3D structural development, and a controlled size and crystallinity [110]. The synthesis involves precipitation and anisotropic growth of MoO_x_-amine precursors formed during the reaction between the molybdenum salt and amine molecule in an acidic environment. The intercalated amine molecule plays three role as a reducing agent, structure-directing agent, and carbon resource [111]. Among the amines used for precursor formation, there are aniline [110,111,112], dopamine, glycine, dodecyl amine, imidazole, 4-Cl-o-phenylenediamine [113], 1,6-hexanediamine, 1,6-hexandiamine, and 1,2-dodecanamine [110]. The precipitated complex is further subjected to thermal decomposition via pyrolysis, where a solid-state reaction occurs with the formation of a metal carbide. The ratio of molybdenum atoms to amine is crucial in the sense of final catalysts. The amine precursor is consumed as much as possible during carbonization, while the unreacted carbon source is a thin graphene/graphite layer on the carbide surface [112]. 

Among factors determining the properties of molybdenum carbides obtained using an amine-metal oxide composite are the temperature of carbonization and the ratio of Mo:amine. Wan et al. [110] synthesized a series of molybdenum carbide powders using various amines including mesitylamine, 4-Cl-o-phenylelediamine, o-phenylenediamine, p-phenylenediamine, aniline, 2-nitro-p-phenylenediamine, 1,6-hexandiamine, 1,12-dodecaneamine, and hexamethylenetetramine. They investigated the effect of properties of the structure-directing agent and temperature of pyrolysis on the crystallinity and morphology of the obtained carbides. They observed that when the Mo:amine (Mo:A) ratio was <1.5:1, β-Mo_2_C was formed, while when Mo:A was equal to or greater than 2:1, the rock salt-type structure of α-MoC_1-x_ was formed. The properties of the amines affected the shapes of carbide particles. For instance, mesitylamine with three methyl groups at meta-positions of an aryl-amine ring directed the formation of nanospherical β-Mo_2_C, while the use of 4-Cl-o-phenylenediamine led to the formation of micro-flowers of rolled-up nanosheets.

The structure-directing method of molybdenum carbide particle preparation may involve other groups of organic precursors such as dyes [114], saccharides [115,116], chelates [117], urea [118,119], metal–organic frameworks [120], polymers (polyaniline, polypyrrole) [121], and volatile organic compounds (VOCs) [122]. Recently, Gavrilova et al. [123] reported a Mo_2_C preparation method via thermal decomposition of molybdenum blue nanoparticles. For the preparation of molybdenum blue, glucose and hydroquinone were used as a carbon source and reducing agent, respectively. Dried molybdenum blue xerogels were calcined at 900 °C in a nitrogen atmosphere. As a result, β-Mo_2_C contaminated with η-MoC,C and γ-MoC was obtained using the method with glucose and hydroquinone, respectively. Mondal et al. [115] reported a method of Mo_2_C incorporated on carbon nanosheets using glucose, heptamolybdate tetrahydrate, and ammonium carbonate. The solution was then dried using spray drying and calcined under nitrogen atmosphere. As a final product, Mo_2_C particles of 5–15 nm crystalline size were obtained on carbon nanosheets. In Table 6, selected methods for molybdenum carbide preparation using the structure-directing method are summarized. Koizumi et al. [122] reported the synthesis of interconnected molybdenum carbide phase nanoflakes that form 3D origami-like structures. The methods for preparation of the structures involved chemical vapor deposition (CVD). As molybdenum and carbon sources, molybdenum trioxide and xylene were used. The synthesis was carried out under Ar:H_2_ (85:15) atmosphere at 790 °C for 1 h. As a result, β-Mo_2_C and graphitic/amorphous carbon phases were obtained.

In the case of TiC, a core–carbon shell structure can also be obtained. However, there are few studies in the literature on the production of titanium carbide with the precursor method compared to the number of articles on tungsten and molybdenum carbides. Gou et al. [124] synthesized a sub-micrometer TiC powder by roasting TiO_2_ encapsulated in phenolic resin, which provided pyrolytic carbon for carbothermic reduction after decomposition at high temperatures (1100–1600 °C). Bae et al. [125] proposed an economical method of producing titanium carbide using precursors, TiO_2_ (P-25) as a source of titanium, and sucrose as a source of carbon. Titanium oxide was suspended in an aqueous saccharide solution and then dried, whereby the TiO_2_ core–sucrose shell precursor was formed. Heat treatment of the obtained precursor at a temperature of 1600 °C in a flowing argon atmosphere allowed obtaining TiC with a specific surface area equal to 137 m^2^/g and an oxygen content of 0.42 wt.% [125]. However, the temperature of the proposed process is still relatively high. Shin and Eun [126] proposed a lower-cost alternative. Metatitanic acid (MTA) and sucrose were used as precursors. MTA is a hydrated titanium oxide of mesoporous nature and is characterized by a lower price than TiO_2_, a large specific surface area of about 337 m^2^/g, and an average pore size of 3.8 nm [127]. The mesoporosity of MTA allows impregnating molecules, e.g., saccharides, into mesopores in aqueous solutions. As a result, a suitable close contact is ensured between the titanium and carbon sources necessary for the synthesis of TiC [126]. However, this method requires sintering in a tube furnace at high temperature (1500 °C) for 2 h under a flowing argon atmosphere. Yu et al. [128] applied polymeric precursors to synthesize titanium carbide fibers. Polytitanoxane (PTO) as the titanium source and polyvinylpyrrolidone (PVP) as the external carbon source and spinning assistant were used in the electrospinning of a precursor process. The prepared precursor fibers were heated at 800 °C for 1 h and then in the range of 1200–1600 °C for the next 1 h [128]. The main disadvantage of this method is the long preparation time of the precursors and the need to use a high temperature.

**Figure 2:**
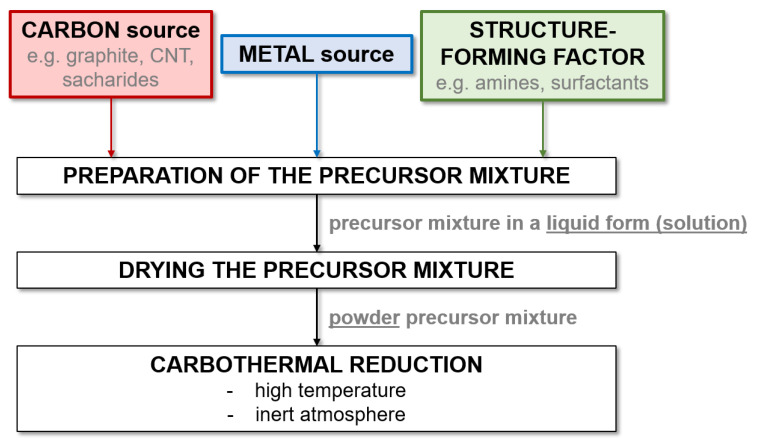
Scheme showing the general steps of the precursor (structure-directing agent) method used to synthesize metal carbides.

When analyzing the data contained in Table 5 and Table 6, it can be seen that the literature contains much more work on the precursor method than research using mechanical milling for the synthesis of W and Mo carbides. This is due to the significantly shorter process time and greater possibilities of controlling the characteristics of the obtained product. This can be achieved both by adding structure-forming substances and by manipulating the process parameters. However, the precursor method requires the use of high temperatures, which is its main limitation. This is most problematic for Ti carbide, as the highest temperatures of all the carbides discussed are required. Therefore, the precursor method is not widely used for the synthesis of TiC. Figure 2 presents a general diagram showing the steps of the precursor method used to synthesize metal carbides.

### 3.4. Molten Salt Synthesis

**Figure 3:**
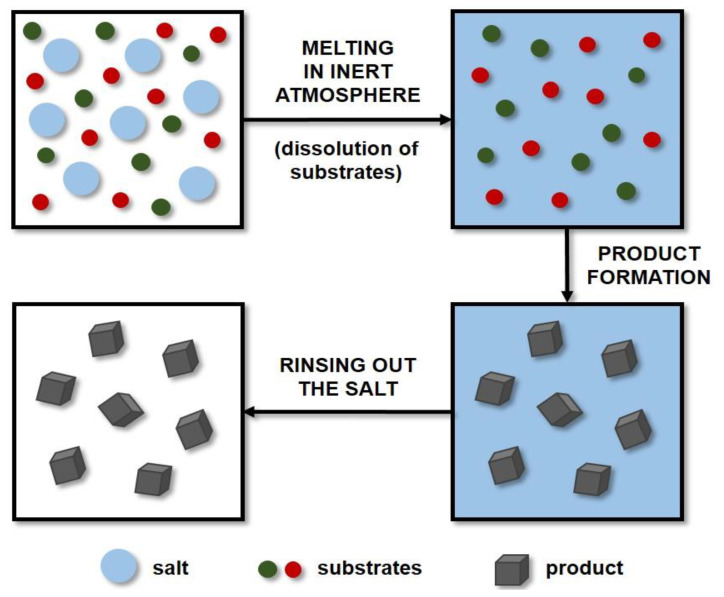
Scheme of molten salt synthesis (MSS).

Molten salt synthesis (MSS) is a well-established and inexpensive technique that has been used extensively in the preparation of ceramic powders. Molten salts are applied as additives to increase the rate of reaction in the solid state. This method uses molten salt as a reaction medium (solvent) to produce the desired product and control its characteristics (including particle shape and size). The reactants are mixed with the salt and then dissolved in it by the action of high temperature. A diagram showing the principle of the MSS method is presented in Figure 3. The amounts of salts used are large, and the most frequently applied systems are eutectic mixtures of chlorides, e.g., NaCl-KCl, and LiCl-KCl, or sulphates, e.g., Li_2_SO_4_-K_2_SO_4_ [140]. The literature includes studies on the production of certain carbides such as titanium carbide [141,142,143], titanium carbonitride [143], or chromium carbide [144] by molten salt synthesis. However, only a small amount of research on the MSS of tungsten carbide has been described thus far.

Yang et al. [145] proposed a procedure for the synthesis of tungsten carbide by the MSS method. As substrates, powder tungsten and activated carbon (200 mesh) were used, which were ground in a high-energy ball mill at room temperature under an argon atmosphere. The ball-to-powder weight ratio was 5:1, and the rotation speed was 1200 rpm. After the grinding process, an equimolar mixture of NaCl and KCl (60 wt%) was mixed with the mechanically activated powder mixture of W and C, and the samples prepared in this way were formed into tablets. The tablets were dropped into molten salt at 1000 °C for 60 min and then air cooled to ambient temperature. After being treated in molten salt, the reaction products were separated by washing with distilled water. In this way, a mixture of WC and W_2_C particles with a diameter ranging from 300 to 500 nm was obtained. A longer milling time resulted in W_2_C gradually converting to WC.

Another approach to the synthesis of WC by the MSS method was presented by Zhang et al. [146]. Pre-dried NaCl and KCl salts were uniformly mixed in a 1:1 molar ratio. Then, a mixture of WO_3_ and graphite particles in different molar ratios was combined with the NaCl-KCl mixture in a weight ratio of 1:30. The powder mixture was heat treated at 950–1150 °C for various times in an argon flow in a tube furnace. After cooling to room temperature, the solidified product was washed with hot distilled water and filtered to remove residual salt, which was repeated several times until no Cl^−^ ions were detected in the filtrate. The products obtained were dried in an oven at 120 °C for 5 h. The proposed method allows obtaining continuous WC coatings consisting of many submicron grains on the surface of graphite particles, and not pure tungsten carbide. The optimal conditions for this process are a heat treatment at 1100 °C for 60 min and a WO_3_/graphite molar ratio in the range of 1:15 to 1:5.

In the work of Qiu et al. [147], WO_3_, carbon black, and NaCl were used as starting materials for the preparation of WC. WO_3_ and carbon black were mixed in a 1:4 molar ratio in a ball mill for 12 h, and then salt in a 1:1 weight ratio of salt:mixture was added. Ethyl alcohol was then added to form a slurry, followed by another 12 h of ball milling. The obtained slurry was completely dried at 50 °C. After the resulting powder had been ground, it was sieved through a 40-mesh screen and calcined at various temperatures for 2 h. After cooling to room temperature, the samples were collected and washed several times with deionized water to ensure that no salt remained before the residual carbon was washed with methylbenzene. The samples were then dried at 60 °C for 24 h. The tungsten carbide plate particles were obtained at a temperature higher than 1200 °C.

Analogous to tungsten carbides, the synthesis of molybdenum carbide with the use of molten salt requires a reaction between molybdenum and carbon in the molten salt medium. Mo and C powders were previously mechanically activated by milling [148,149]. Yang et al. [148] synthesized Mo_2_C using the MSS method. Firstly, Mo and C powders were mechanically activated under an argon atmosphere using a high-energy planetary mill (10 mm hardened steel ball, ball-to-powder ratio: 5:1, rotational speed 1200 rpm). After milling, activated powder was blended with a KCl and NaCl (1:1) mixture and annealed at 1000°C for 60 min. After, the reaction product was heated to room temperature under ambient air. Finally, salts were removed by washing with water. The final product consisted of molybdenum carbide Mo_2_C with a particle size of 0.5–1.0 um contaminated with molybdenum metal. As a starting material for the preparation of molybdenum carbide using molten salt synthesis, MoO_3_ has been used. Hu et al. [150] described the method of preparation of molybdenum carbide doped with nickel supported on carbon fiber paper (CFP). For synthesis, CFP was impregnated with nickel ions. Then, molybdenum trioxide was mixed with carbon black and salt (NaCl and KCl) particles. Finally, the impregnated CFP and MoO_3_/carbon black/KCl/NaCl were combined together and annealed at 1000 °C for a 309 h period. The reaction in molten salt can be carried out while using other techniques [151,152,153]. Ge et al. [153] reported the electrochemical method of Mo_2_C preparation using Mo, Pt, and C as a cathode, anode, and counter electrode, respectively. The reaction between Mo and C was carried out in the molten salt of LiCl, NaCl, and Na_2_CO_3_ at 900 °C. The final product was composed of carbon films with Mo_2_C interlayers. Recently, an electrochemical method that uses carbon dioxide as a source of carbon has been gaining increasing interest due to the possibility of utilizing one of the main greenhouse gases [151,152]. Chen et al. [152] reported a new method for the preparation of molybdenum carbide combining the MSS and the electroreduction methods. In this method, carbon dioxide was reduced on a Mo cathode to form molybdenum carbide. The set-up comprised molybdenum foil and tin oxide (SnO_2_) as a cathode and anode, respectively. The electrodes were immersed in a crucible filled with a mixture of CaCl_2_ and CaO and heated under a CO_2_ atmosphere up to 850 °C for 5 h. The obtained results indicated the formation of MoC and Mo_2_C crystal phases with a lamellar structure. Before both non-electrochemical and electrochemical synthesis, the preliminary preparation of salts is required. The salts have to be thermally treated in two steps. The first step refers to the removal of the moisture absorbed by the salts (200–400 °C), and the second step (the synthesis reaction) refers to the removal of oxygen from the pores and the intergranular space. The steps are processed under air and Ar, respectively [154,155,156].

In the case of titanium carbide, the formation and growth of crystals in the molten salt occur mainly through the “carbon template growth” mechanism [154,157]. Ti powder dissolves in molten salt and then adheres to the surface of the carbon source. TiC formation occurs from the surface to the inside of the carbon [154,155]. Yang et al. [156] described the following mechanism for forming TiC powder in the molten salt system. The first step is the dissolution of a small amount of Ti powder, which leads to the formation of Ti (II) and Ti (III) species. After that, Ti (II) moves towards the surface of the carbon source, which is floating on the surface of the molten salt. As a consequence, the in situ synthesis of nanocrystalline TiC occurs according to the reaction presented by Equation (1). The titanium atoms come from the disproportionation reaction of Ti (II) in the molten salt, and C atoms on the surface of the carbon sources react. Meanwhile, dissolved Ti (III) species react with the titanium powder to form Ti (II) until the Ti powder is completely burned out [156].

(1) $$3Ti\left(II\right)+C\;\overset{\;\;\;\;\;\;\;\;\;\;\;\;\;\;\;}{\to }\;TiC+2Ti\left(III\right)$$

The selected process parameters for TiC synthesis using the molten salt synthesis method and the characteristics of the obtained particles are summarized in Table 7. All processes presented were performed under a flowing argon atmosphere. Table 8 presents the parameters of the selected molybdenum carbide preparation processes based on the MSS method. The majority of reports in the literature describing the use of this method refer to the synthesis of molybdenum and titanium carbides. For WCs, only a few studies are available. However, the MSS method is gaining increasing popularity in the synthesis of metal carbides, especially in the case of titanium, as it allows the process temperature to be significantly reduced compared to that of other methods. Moreover, the required process times are relatively short. Other advantages of MSS methods are certainly the low cost of the salts used and the ability to control the characteristics of the obtained product. However, research often concerns obtaining carbide coatings on the surface of a carbon template, and not a pure metal carbide. Furthermore, this method also has the disadvantage of having to work with easily oxidizable metal powders, meaning that all handling of these reagents must be carried out under an inert gas atmosphere, which complicates the process.

Independently of the preparation procedure, due to the pyrophoric nature of metal carbides, it is necessary to stabilize the carbide structure via passivation. Mehdad et al. [74] investigated the effect on passivating agents of molybdenum and tungsten carbides on catalytic activity in the toluene hydrogenation process. As passivating agents, they examined carbon dioxide, water, and oxygen. They found that CO_2_ and H_2_O require high temperatures, 505 and 580 °C for H_2_O and CO_2_, respectively, to dissociate and react with the carbide surface. Moreover, at high temperature, they remove carbon from the catalyst surface. The best passivating agents turned out to be diluted oxygen. In the case of Mo_2_C, using 0.1% allowed for effective passivation and recovery to unpassivated carbide at 300 °C and under a hydrogen atmosphere. Ten times higher oxygen concentrations resulted in a passivated structure that requires higher temperatures to recover (650 °C). In the case of WC, no significant differences were observed between samples treated with different oxygen concentrations [74]. 

## 4. MAX Matrices and MXenes

A special family of transition metal carbides is constituted by multilayer metal carbides with a 2D nanosheet structure similar to that of graphene, belonging to the group of compounds called MXenes. The term MXenes denotes carbides and nitrides of transition metals, with the general formula *M*_*n*+1_*X_n_T_x_*, where *n* = 1, 2, 3, or 4, *M* refers to the transition metal (*M* = Sc, Ti, V, Cr, Mn, Y, Zr, Nb, Mo, Hf, Ta, and W [23,161]), and *X* refers to the p-block element (silicon, aluminum, gallium), while *T* describes the type of terminal groups (–O, –OH, –F, –Cl) in the amount of *x* per selected unit. They are obtained by selectively removing component *A* from the ternary MAX matrix. The MAX matrix consists of the elements of the transition metal *M*, a *p* group element (A), and carbon or nitrogen (X). MXene compounds are gaining importance due to their metal-like conductive properties, thermal and chemical stability, and the ability to manipulate properties through simple and effective modification of terminal groups [162,163]. Their unique properties allow for application in various branches of science: energy storage [163,164], electrocatalysis [165,166], photocatalysis [167,168], and heterogeneous catalysis [23,164,165,166,169].

Recently, a new group of MAX matrix compounds was discovered named i-MAX. The i-MAX family represents quaternary transition metal carbides of the general formula (M^1^_2/3_M^2^_1/3_)_2_AlC, where the M^1^ and M^2^ elements are two different early transition metals with in-plane ordering. The i-MAX phases exhibit orthorhombic symmetry, where M^2^ extends M^1^ planes out towards A element layers [165,166].

### 4.1. Synthesis of MAX Matrices

The synthesis of MAX matrices involves high-temperature sintering of elements, leading to a solid-state reaction. In Table 9, selected methods of the synthesis of MAX matrices are presented. Undoubtedly, the vast majority of the literature reports on titanium aluminum carbides or their combination with other MAX matrices. The first step of the synthesis is the preparation of the reactants by grinding (milling) them into smaller sizes to develop the contact surface area. The grinding (milling) process may be carried out dry or wet. Dry milling is carried out in an inert atmosphere to avoid partial oxidation of the substrates. Wet milling may be carried out in organic media such as alcohols (ethanol, propanol) and liquid hydrocarbons (heptane) [15]. In some literature reports, mixing substrates in an alcoholic solution without the grinding step is also described [170,171]. As substrates, mainly powders of constituent elements are used. The molar ratio of the transition metal and the carbon source corresponds to the molar ratio in the final compound. In many cases, the excess of volatile ingredients (Al, Ga, Si) is used due to the possible loss of this element during high-temperature treatment, flow of inert gas, and conversion to oxides by reaction with oxygen contamination from starting materials [172,173]. The temperature of the solid-state reaction ranges from 800 up to 1600 °C, depending on the MAX matrix. MAX matrices are contaminated with intermetallic compounds or metal oxides; therefore, it is necessary to remove these impurities by dissolution in concentrated acids, for example, H_3_PO_4_ and HCl [162,174,175,176]. 

The synthesis method using shielded molten salts (MS^3^) is becoming increasingly important. The use of eutectic molten salt systems allows for a reduction in the temperature of the synthesis process and inhibits the oxidation of the forming carbide phase. The melted salts provide a reaction medium and prevent the reacting species from oxidation by forming a protective barrier, impermeable to oxygen molecules present in the air. The solid-state reaction occurs via interdiffusion of atomic species in the molten medium, leading to a dissolution–precipitation process. As a single reaction medium, or as mixtures, sodium chloride, potassium chloride, and potassium bromide [175] have already been used. The selection of particular salts depends on the melting points and the level of pressability to provide a gas-tight shell [175]. After synthesis, the MAX matrix is separated from the salts by washing with water. Recent findings indicate that it is possible to synthesize MAX matrices using the MS^3^ method under air atmosphere [175,177].

### 4.2. Synthesis of MXenes

Multilayer (accordion-like) or single-layered transition metal carbides called MXenes are obtained using the selective etching method. MXenes are obtained via selective etching of the A element from the MAX matrix. MXenes obtained from i-MAX matrices are characterized with the M_1.33_X formula. During the etching of M^2^, the early transition metal is also removed [165,166]. As a result of etching, MXene sheets are covered with terminal groups –F, –O, and/or –OH. The metallic bonds between the M and A elements are replaced by hydrogen and van der Waals bonds.

As an etchant, the most common used is concentrated hydrofluoric acid, which is an in situ formed hydrofluoric acid. In situ HF is formed by mixing concentrated hydrofluoric acid and fluorine salt (LiF, NaF, KF, NH_4_HF_2_) [164,178]. The use of in situ methods avoids the challenges of working with and maintaining harmful HF. Moreover, the use of this method allows simultaneous deflection and ion intercalation in one step [174,179]. The temperature, time, and concentration of the etchant affect the structure of the MXene. Depending on the MAX phase, the etching may lead to structures from multilayer lamellas to densely packed particles. The etching of Al from the MAX structure is carried out mainly at room temperature; however, in the case of molybdenum aluminum carbide (Mo_2_Ga_2_C) and vanadium aluminum carbide (V_2_AlC), etching at higher temperature equal to 55 °C and 90 °C, respectively, was reported [169,174,180]. Selection of appropriate etching conditions is crucial in terms of physicochemical properties, including the size and distribution of sheets determining its application. Conditions that are too mild may not lead to complete leaching of element A, while conditions that are too aggressive may lead to over-etching of element M and the formation of a large number of surface defects [181]. 

After the etching process, the remaining material should be washed with water to neutralize it until pH = 6. The next step in the processing of an MXene into a single-flake morphology is an intercalation. As intercalates, dimethyl sulfoxide (DMSO), isopropyl alcohol, and an aqueous solution of base intercalants, such as tetrabutylammonium hydroxide (TBAOH, 40 wt% in H_2_O) and tetrapropylammonium hydroxide (TPAOH, 40 wt.% in H_2_O), are used. When using an in situ HF-formed etchant, intercalation proceeds at the etching step due to the presence of Li^+^, NH_4_^+^_,_ or K^+^ ions. In the case of the basic intercalant, the MXene phase is further washed to obtain a pH below 8 [171,182,183].

In high temperatures and an oxidizing environment, MXene compounds may be partially or completely oxidized to metal oxides, which will lead to a loss of their properties. Partial oxidation occurs even though the high-temperature treatment is carried out under an inert atmosphere (Ar or N_2_) due to dehydration and removal of intercalated species and terminal groups (Equation (2)). As a result, partial oxidation with released water occurs, leading to the formation of an oxycarbide phase [184,185].

(2) $$M_{n+1}X_{n}{\left(OH\right)}_{2}\overset{\;\;\;\;\;}{\to }M_{n+1}X_{n}O+H_{2}O$$

### 4.3. Modification of MXenes with Other Compounds

Recently, an increasing number of research reports on modified metal carbides from the MXene group to improve catalytic [169,180], conducting [186,187], photocatalytic [167,182], and optical response properties [167,182] have been published. He et al. [187] synthesized Ni_1.5_Co_1.5_S_4_@Ti_3_C_2_ nanocomposites for high-performance supercapacitors. Metal sulfides were obtained by co-precipitation from chloride salts with thiourea and sodium hydroxide (pH = 10). The deposition of metal sulfides on the MXene surface resulted in enhancement of conducting properties. The deposition of metal ions on the MXene surface proceeds through electrostatic adsorption of metal ions from the negatively charged MXene surface. Another research group obtained a nickel-modified MoO_2_@Mo_2_CT_x_ nanocomposite. Nickel ions were deposited on Mo_2_CT_x_ from an aqueous nitrate solution. The sample was then calcinated in an argon atmosphere to protect it from oxidation and annealed at 450 °C in an atmosphere of H_2_/Ar. The obtained results of the catalytic activity of the supported nickel catalysts showed increased activity and stability in the hydrodeoxygenation of palmitic acid reaction. Improved catalytic activity was attributed to the synergetic effect between the nickel, Mo_2_CT_x_, and MoO_2_ catalytic sites [169]. The upgraded properties of metal/metal oxide/MXene nanocomposites were also observed for Fe/TiO_2_/Ti_3_C_2_ photocatalysts. Grzegórska et al. [167] obtained TiO_2_/Ti_3_C_2_ nanocomposites by hydrothermal treatment of the Ti_3_C_2_ MXene. As a result, decahedral TiO_2_ of exposed {101} and {001} facets created a highly efficient connection in the photocatalytic degradation of pharmaceuticals. The photocatalytic activity was further improved via deposition of iron particles on the nanocomposite using a magnetron sputtering system. 

## 5. The Use of Metal Carbides for Dry Reforming

### 5.1. Tungsten Carbide

Compared to noble metals, nickel-based catalysts have a lower cost; therefore, they are commercially used in the methane reforming [190]. However, their major disadvantage is that during the decomposition of CH_4_ and CO disproportionation, they can be deactivated by forming coke [191,192]. Therefore, alternative catalysts are sought, the use of which would eliminate the existing problems. Transition metal carbides such as WC have been shown to be good catalytic materials. They have a very high catalytic activity (at a level similar to that of noble metals such as Ru and Pt) [27], are stable and highly selective in a wide range of reactions, and are also resistant to the presence of contaminants such as sulfur and chlorides in the reaction medium. They are also highly resistant to carbon deposition [193]. Generally, the order of stability of group V and VI transition metal carbides in the case of methane dry reforming is as follows: Mo_2_C ≈ WC > VC > NbC > TaC, at the reaction pressure of 8 bar, and Mo_2_C > Ir > WC > Pd >Pt, in the case of 2 bar [27,69]. Therefore, it should be noted that carbides of the Mo_2_C and WC types show stable activity only at relatively high pressures [69]. At atmospheric pressure, a significant limitation is the deactivation of such catalysts through oxidation with carbon dioxide [194], which occurs through dissociative CO_2_ adsorption and oxidation of the carbide with oxygen atoms [195]. Thus, the stability of the catalyst is determined by the ability to convert the oxide back to carbide, which is assisted by high temperatures. Consequently, tungsten or molybdenum carbide can act as redox catalysts in dry methane reforming, but it is worth bearing in mind that at atmospheric pressure, the reaction of CO_2_ with this type of carbide is more favorable than the reaction of CH_4_ with the oxides formed [69].

Comparing the phases of WC that occur, it may be concluded that the hexagonal close-packed β-W_2_C is the most active, while the hexagonal α-WC is slightly less active, and the fcc WC_1-x_ is twofold less active [196]. β-W_2_C nanoparticles are characterized by a disordered structure and the presence of carbon vacancies [28], as a result of which they have greater stability compared to α-WC nanorods [196,197]. According to research conducted by Zhang et al. [198], during dry methane reforming, oxidation of β-W_2_C by CO_2_ readily occurs, resulting in phase transformation to α-WC. The following steps of β-W_2_C oxidation can be described by Equations (3)–(6).

(3) $$\beta -W_{2}C+3CO_{2}\overset{\;\;\;\;\;\;\;\;\;\;\;\;\;\;}{\to }WO_{2}+4CO+W$$

(4) $$WO_{2}+CH_{4}\overset{\;\;\;\;\;\;\;\;\;\;\;\;\;\;}{\to }\alpha -WC+2H_{2}O$$

(5) $$2WO_{2}+CH_{4}\overset{\;\;\;\;\;\;\;\;\;\;\;\;\;\;}{\to }\beta -W_{2}C+4H_{2}O$$

(6) $$W+CH_{4}\overset{\;\;\;\;\;\;\;\;\;\;\;\;\;\;}{\to }\alpha -WC+2H_{2}$$

However, according to Yan et al. [199], two-sided reactions are possible when using tungsten carbide in the dry methane reforming process. The first reaction is the oxidation of WC by CO_2_ (Equation (7)), and the second is the reverse gas–water shift reaction (Equation (8)).

(7) $$WC+CO_{2}\overset{\;\;\;\;\;\;\;\;\;\;\;\;\;\;}{\to }W+2CO$$

(8) $$H_{2}+CO_{2}\overset{\;\;\;\;\;\;\;\;\;\;\;\;\;\;}{\to }CO+2H_{2}O$$

In addition, an increasing amount of research is currently focused on discovering new methods of tungsten carbide synthesis leading to various types of morphologies such as nanoparticles [200], nanosheets [69], and nanorods [201]. The results of these studies indicate a definite relationship between the characteristics of the obtained particles and the catalytic activity. However, there are very few works that try to explain this relationship. This may be due to significant synthetic limitations, especially since a large part of this research is not experimental [202,203]. 

### 5.2. WC Combined with Nickel and Cobalt Particles

Tungsten carbide is used as a catalyst in dry methane reforming, usually in combination with nickel or cobalt, because the addition of a second metal can modify the catalytic performance and structure of this carbide [204,205]. Despite the unique properties of WC, this compound has a surface with a strong oxygen affinity. As a consequence, this leads to blockage of the surface in the event of irreversible adsorption of oxygen-containing substances, which, in turn, results in a reduction in catalytic activity [206]. Therefore, to avoid this problem, core–shell systems are used, that is, WC cores covered with a metallic coating that prevents oxidation of the carbide surface, thus promoting structural stability [207]. Co-WC and Ni-WC are stable, active, and selective catalysts in dry methane reforming [208]. In a study by Yao et al. [69], it was shown that the Ni-WC catalyst has higher stability than Ni-Mo_2_C due to tungsten carbide’s sintering resistance [28], which was observed with a similar size of crystallites before and after the reaction. Additionally, Ni-WC is also resistant to oxidation during the process, unlike the Ni-Mo_2_C catalyst. 

According to Barbosa et al., higher CO_2_ conversion values compared to CH_4_ conversion in dry methane reforming are obtained using Ni-Mo_2_C and Ni-WC catalysts [209]. This is probably due to the reactions occurring, including the Boudouard reaction (Equation (9)), as a result of which the forming CO_2_ is activated in the carbide (Equation (10)), leading to the oxidation of WC (Equations (11) and (12)) and the conversion of CO with steam (Equation (13)), in which part of the hydrogen obtained reacts with CO_2_, resulting in a lower H_2_/CO ratio and in increased CO_2_ conversion. However, regardless of the presence of nickel and the Ni/W ratio, the less stable β-W_2_C is transformed into α-WC during dry methane reforming, according to Equations (3)–(6) [198].

(9) $$2CO\overset{\;\;\;\;\;\;\;\;\;\;\;\;\;\;}{\to }CO_{2}+C_{\left(s\right)}$$

(10) $$CO_{2}\overset{\;\;\;\;\;\;\;\;\;\;\;\;\;\;}{\to }CO+O^{*}$$

(11) $$CO_{2}\overset{\;\;\;\;\;\;\;\;\;\;\;\;\;\;}{\to }CO+O^{*}$$

(12) $$WC+3O^{*}\overset{\;\;\;\;\;\;\;\;\;\;\;\;\;\;}{\to }WO_{2}+CO$$

(13) $$CO+H_{2}O\overset{\;\;\;\;\;\;\;\;\;\;\;\;\;\;}{\to }CO_{2}+H_{2}$$

In the case of cobalt tungsten carbide (Co_6_W_6_C), the addition of carbon in the early stages of the catalytic reaction results in the conversion of the bimetallic carbide to a stable form containing active sites for dry methane reforming [205], according to Equation (14).

(14) $$Co_{6}W_{6}C+5C\overset{\;\;\;\;\;\;\;\;\;\;\;\;\;\;}{\to }6Co+6WC$$

Furthermore, Shao et al. [205] investigated the effect of temperature on the performance of cobalt-tungsten carbide as a catalyst for dry methane reforming. It was found that at too low process temperatures (500–850 °C), the oxygen on the bimetallic carbide was converted to an ineffective metallic oxide, which can be described by Equation (15). Meanwhile, in the case of the process carried out at appropriate high temperatures (above 850 °C), CH_4_ decomposed and formed decomposition products, removing the surface oxygen or oxide formed, according to Equation (16), which resulted in obtaining an active and stable phase for the reforming, containing Co, WC, and C in the bulk phase.

(15) $$Co_{6}W_{6}C+\left(26-x\right)O_{\left(surface\right)}\overset{\;\;\;\;\;\;\;\;\;\;\;\;\;\;}{\to }6CoWO_{4}+xCO+\left(1-x\right)CO_{2}$$

(16) $$CoWO_{4}+2CH_{4}\overset{\;\;\;\;\;\;\;\;\;\;\;\;\;\;}{\to }Co+WC+C+4H_{2}O$$

Table 10 summarizes the parameters of the selected dry methane reforming processes and the results obtained with the use of tungsten carbide-based catalysts.

### 5.3. Molybdenum Carbide

Molybdenum carbide is the most widely used transition metal carbide. In the dry reforming of hydrocarbons, Mo_2_C exhibits superior catalytic activity and stability. The mechanism of dry reforming of methane is based on the oxidation–recarburization cycle and noble metal-type mechanisms [82,83,124,199,215], as presented in Equations (17)–(26).

(17) $$Mo_{2}C+5CO_{2}=2MoO_{2}+6CO$$

(18) $$2MoO_{2}+5CH_{4}=Mo_{2}C+4CO+10H_{2}$$

(19) $$MoO_{2}+2H_{2}=Mo+2H_{2}O$$

(20) $$2Mo+CH_{4}=MoC_{2}+2H_{2}$$

(21) $$Mo_{2}C+5H_{2}O=2MoO_{2}+CO+5H_{2}$$

(22) $$H_{2}+CO_{2}=CO+H_{2}O$$

(23) $$CH_{4}=C+4H$$

(24) $$2C+3MoO_{3}=3MoO_{2}+CO_{2}+CO$$

(25) $$2H+MoO_{3}=MoO_{2}+H_{2}O$$

(26) $$3C+2MoO_{2}=Mo_{2}C+2\;CO+CO_{2}$$

An overwhelming number of research reports on the catalytic activity of molybdenum carbides in the dry reforming of hydrocarbons refer to catalysts prepared using TPR method. The physicochemical properties and resulting catalytic activity of molybdenum carbide catalysts are influenced by the molybdenum-to-carbon ratio. Gao et al. [79] reported a series of molybdenum carbide catalysts that differ in the weight content of Mo in order to use carbon nanotubes as a carbon source (Mo 0, 5, 10, 15, 30, 60, and 100 wt.%). Along with an increasing proportion of molybdenum in the catalyst, a decrease in the specific surface area, diameter, and pore volume was observed. A correlation was observed between the molybdenum content and catalytic activity in the dry methane reforming process. The highest activity was observed for the catalyst containing 30 wt.% of Mo. Another of the key structural parameters of the carbide for catalytic activity is the excess unbound carbon formed during the synthesis process. Roohi et al. [216] found that the amount of excess carbon depends on the carburization temperature and the concentration of carbon-containing gas during the synthesis. Catalysis with lower contents of excess carbon exhibited an initial higher activity in the DRM reaction; however, during the long-term test, the molybdenum loading was a crucial factor. 

Several articles have been published to investigate the effect of the crystal structure on catalytic activity in DRM [78,217]. Liang et al. [78] investigated the catalytic activity of β-Mo_2_C and α-MoC_1-x_ phases in DRM. Both phases were characterized by a narrow size distribution of up to 5 nm. Better activity was observed for the -MoC_1-x_ phase. Oshikawa et al. [217] observed the dependence of the η-Mo_3_C_2_ phase on the methane decomposition rate. They reported the key role of the η-Mo_3_C_2_ phase among other molybdenum carbide phases as an active species for methane reforming. During the DRM process, the molybdenum carbide may be partially oxidized to the form of an oxycarbide. Kurlov et al. [218] reported that the oxycarbidic phase Mo_2_C_x_O_y_ exhibits high stability toward further oxidation to MoO_2_, and the increase in β-Mo_2_C/ Mo_2_C_x_O_y_ active sites correlates with higher efficiency in the DRM reaction. 

Bulk molybdenum carbide is characterized by a low surface area. To develop the surface area and improve the stability and activity before the TPR process, a molybdenum precursor is deposited as a support. As a support, γ-Al_2_O_3_ [80,81,143,215,219], ZrO_2_ [80,81,143], MgO [76], zeolite beta [138], SiO_2_ [75], and TiO_2_ [75,138] have been reported. The role of the support is to preserve a high dispersion of molybdenum carbide. Darujati et al. investigated the stability of a Mo_2_C supported catalyst synthesized using the TPR technique (20%CH_4_/H_2_, 675°C). They found that low-surface area supports (MgO: 37 m^2^/g, and α-Al_2_O_3_: 3 m^2^/g) caused deterioration of the catalytic properties. Supports with a high surface area (ZrO_2:_ 102 m^2^/g, y-Al_2_O_3:_ 200 m^2^/g) improved the catalytic activity; however, in the case of ZrO_2_, rapid support sintering occurred. The best activity was observed for Mo_2_C/y-Al_2_O_3_ catalysts. The superior activity was attributed to the strong interaction of MoO_3_ and Al_2_O_3_ that led to the formation of a molybdenum monolayer, which preserved a high Mo_2_C dispersion. Brungs et al. [75] obtained similar results. They investigated aluminum oxide (194 m^2^/g), zirconium oxide (90 m^2^/g), titanium dioxide (150 m^2^/g), and silicon oxide (320 m^2^/g) as supports for a Mo_2_C catalyst prepared using carburization (10% *v/v* C_2_H_6_/H_2_, 900 K) of MoO_3_ supported with the selected metal oxides. The catalysts were ranked in order of decreasing activity and stability: Mo_2_C/Al_2_O_3_ > Mo_2_C/ZrO_2_ > Mo_2_C/SiO_2_ > Mo_2_C/TiO_2_. The highest activity of the Al_2_O_3_- and ZrO_2_-supported catalysts was attributed to the formation of the MoO_3_ monolayer during a short calcination period. 

Molybdenum carbide catalysts were also tested for the dry reforming of hydrocarbons other than methane. Dry reforming of ethane (DRE) proceeds through reduction of ethane and oxidative dehydrogenation (ODH), leading to the formation of ethylene. The processes taking place are illustrated by Equations (27)–(28) [220]:

(27) $$2CO_{2}+C_{2}H_{6}=4CO+3H_{2}$$

(28) $$CO_{2}+C_{2}H_{6}=C_{2}H_{4}+CO+3H_{2}O$$

Dry reforming of ethane differs from DRM in order to obtain the H_2_/CO composition in the outlet stream. The H_2_/CO ratio is lower than that of DRM, and it very often oscillates around 0.5. Porosoff et al. [220] investigated the catalytic activity of Mo_2_C/Al_2_O_3_ in the dry reforming of ethane. It was found that carbide catalysts promoted the formation of ethylene rather than the production of syngas through the DRE path. Dry reforming of hydrocarbons with alkyl chains longer than C_2_ requires milder conditions than those for methane [220]. Carbon dioxide oxidizes the surface of Mo_2_C catalysts to produce oxycarbides. The conversion of hydrocarbons with carbon chains longer than C_2_ with carbon dioxide promotes dehydrogenation and aromatization reactions [215,221]. For propane, the oxidative dehydrogenation processes are described by Equations (29)–(33). Propane forms a surface complex with active oxygen from the oxycarbide (29). In the next step, the C-H bond is broken in the reduced centers (30) or another active oxygen (31). As a result of the catalytic reaction of propane with CO_2_ under Mo_2_C catalysts, propylene is mainly formed.

(29) $$C_{3}H_{8}\left(g\right)+O\left(a\right)↔\;C_{3}H_{8}O\left(g\right)$$

(30) $$C_{3}H_{8}O\left(a\right)=C_{3}H_{7}O\left(g\right)+H\left(a\right)$$

(31) $$C_{3}H_{8}O\left(a\right)+O\left(a\right)↔\;C_{33}H_{7}O\left(g\right)+OH\left(a\right)$$

(32) $$C_{3}H_{7}O\left(a\right)=C_{3}H_{6}\left(g\right)+OH\left(a\right)$$

(33) $$2OH\left(a\right)=H_{2}O\left(g\right)+O\left(a\right)$$

### 5.4. Molybdenum Carbide Modified with Nickel Particles

Molybdenum carbide catalysts during DRM at atmospheric pressure may suffer from deactivation due to oxidation with carbon dioxide. The carbide structure is reconstructed with the carbon element from the dissociation of methane; however, oxidation with CO_2_ is more favorable [219]. The combination of molybdenum carbide with other metals: Ni [222], Co [223], and Fe [24], allows controlled dissociation paths of CO_2_ and CH_4_, ensuring appropriate conditions for oxidation–recarburization cycles [47,224]. The introduction of other metals into the carbide catalyst results in the generation of more moles of hydrogen, leading to a higher H_2_/CO ratio in the outlet stream. Carbide and the introduced metal (Ni, Co) act as an active center for the dissociation of CO_2_ and methane, respectively. 

It is generally accepted that the catalytic activity of nickel catalysts is strictly connected with the size of the nickel particles: the smaller the Ni particles, the better the catalytic activity, resulting from the stronger active metal–support interactions, delayed sintering, and a lower rate of formation of carbon deposits [3,5,14,225]. However, in the case of molybdenum carbide supported nickel catalysts, the ratio of Ni/Mo to the size of nickel particles plays a predominant role [51,226]. The nickel-to-molybdenum ratio affects the morphology and catalytic activity of Mo_2_C. Moreover, too high a dissociation of CH_4_ promotes the formation of coke on the surface of the catalysts [79,222]. Zhang et al. [222] observed that with an increasing nickel content in nickel-modified Mo_2_C supported on carbon nanotubes, the crystallite size of Mo_2_C for Ni/Mo ratios = 0.5, 1, 1.5, and 2 was equal to 53, 38, 35, and 28 nm, respectively. Moreover, the increase in the Ni content resulted in an increase in the particle size. Catalytic activity increased with an increasing Ni/Mo ratio to the optimal value (1:1). After this value was exceeded, the activity decreased despite the higher content and smaller particle size of nickel. The DRM process is carried out mainly at temperatures above 800 °C. The performance of processes at lower temperatures results in lower methane and carbon dioxide conversions, as well as a lower H_2_/CO ratio [119,123]. However, Diao et al. [227] recently reported the high catalytic activity of a Ni-Mo_2_C/Al_2_O_3_ catalyst at 470 °C in a catalytic bed coupled with non-thermal plasma treatment. The molybdenum-nickel-alumina catalyst exhibited superior activity compared to Ni/Al_2_O_3_. The H_2_/CO ratio was equal to 0.9, and the conversions of CH_4_ and CO_2_ were around 80% and 85%, respectively. 

Both bare and nickel-modified molybdenum carbide catalysts are used, both supported and unsupported. Deposition on an inert substrate allows for dilution of the catalyst, thus eliminating channeling, and retarding heat transfer limitations and pressure drop across the catalytic bed [194]. As a support, metal oxides: La_2_O_3_ [224], Al_2_O_3_ [12,121,226], SiO_2_ [10], ZrO_2_ [116], MgO [228], biochar [77], carbon nanotubes [222], zeolites [26], and silicon carbide [10], have been examined. Silva et al. [10] investigated the effect of the support (SiO_2_, Al_2_O_3_, and SiC) Ni-Mo_2_C on catalytic activity and stability in the DRM reaction. The lowest DRM substrate conversions and H_2_/CO ratios were observed for the silica support. As a reason for the low activity observed for the SiO_2_-supported samples, there were weak interactions between Ni and SiO_2_, leading to movement of Ni species at the surface of the catalysts, retarding the interface contact between Ni and Mo_2_C responsible for the oxidation–recarburization cycle, Ni aggregates, and the formation of filamentous carbon. Table 11 and Table 12 summarize the parameters of the selected dry methane reforming processes and the results obtained with the use of molybdenum carbide and nickel-modified molybdenum carbide-based catalysts, respectively.

Recently, Zhang et al. [222] proposed a new cycle route for Ni/Mo_2_C under dry reforming conditions. They identified MoNi_4_ as an intermediate during the oxidation–carburization process (OCP). The phase is formed during the reaction between MoO_2_ and the generated hydrogen. The carbide phase is reconstructed during the in situ carburization of the reaction. The MoNi_4_ phase was found to be catalytically inactive, and when it is formed in large quantities, it is one of the main factors that causes complete deactivation of the catalyst. The transformations in OCP are given by Equations (34)–(37).

(34) $$Ni/Mo_{2}C+5CO_{2}=2Ni/MoO_{2}+6CO$$

(35) $$2Ni/MoO_{2}+2H_{2}=2Ni/Mo_{\;}+2H_{2}O$$

(36) $$Ni/Mo_{\;}\overset{\;\;\;\;\;}{\to }MoNi_{4}$$

(37) $$2MoNi_{4}+CH_{4}=Ni/Mo_{2}C+H_{2}$$

### 5.5. MAX and MXenes for Dry Reforming of Hydrocarbons 

Despite a broad examination in electrocatalysis, heterogeneous photocatalysis, and catalysis, to our best knowledge, to date, among titanium, molybdenum, and tungsten MXenes or MAX matrices, the catalytic activity in dry reforming of hydrocarbons has been reported only in a few articles. Ronda-Lloret et al. reported the catalytic activity of Co_3_O_4_ supported on Ti_2_AlC in the dry reforming of butane [230]. The levels of butane and carbon dioxide conversions for Co_3_O_4_/Ti_2_AlC were 20% and 25% after 18 h of testing, respectively. The efficiency of butane conversion was higher compared to Co_3_O_4_/TiO_2_; however, it was lower than that for Co_3_O_4_/Al_2_O_3_. Despite the lower activity, the Co_3_O_4_/Ti_2_AlC catalyst exhibited higher stability and anticoking properties compared to the metal oxide-supported catalysts.

Kurlov et al. [231] reported the catalytic activity of a 2D-Mo_2_CO_x_/SiO_2_ catalyst in the dry reforming of methane. The catalyst was prepared by incipient wetness impregnation of multilayered m-Mo_2_CT_x_ on a SiO_2_ support in a colloidal alcoholic suspension, followed by reduction in hydrogen (20 vol.% H_2_/N_2_, 800 °C) and oxidation with CO_2_. The authors found that the deposition on silica particles prevents the thermal sintering and oxidation of the Mo_2_C and MoO_2_ phases, respectively, while activation with CO_2_ is crucial to protect the catalysis from complete oxidation. Furthermore, they found that long-term storage of 2D-Mo_2_CO_x_/SiO_2_ leads to partial fragmentation of nanosheets and thus to deactivation of the catalyst. 

Among others, the catalytic activity of V_2_O_3_−V_8_C_7_/m-V_2_CT_x_, obtained from the V_2_AlC matrix, belonging to the MAX/MXene family, in the dry reforming of methane was reported [226]. The V_2_O_3_−V_8_C_7_/m-V_2_CT_x_ catalyst exhibited catalytic activity comparable to the nickel catalyst supported on ZSM-5 zeolite. After the catalytic process, the remaining V_2_O_3_−V_8_C_7_/m-V_2_CT_x_ catalysts’ layered structure was slightly oxidized into an oxycarbide. The thermal stability at high temperatures, anti-oxidation properties under mild oxidants (CO_2_), and ability to participate in oxidation–carburization cycles are crucial factors in terms of potential application in the dry reforming of hydrocarbons.

## 6. Conclusions and Future Perspectives

Transition metal carbides, mainly tungsten and molybdenum carbides, are an interesting group of compounds that can be used as catalysts in the dry reforming process. Literature analysis shows that such a solution is becoming increasingly popular and is being investigated widely. This is because of their high thermal stability and high catalytic activity. 

TMCs may be prepared via high-temperature reactive sintering, temperature-programmed reduction (TPR) and carburization (TPC), high-energy ball milling, structure-directing methods, and synthesis in a molten salt medium. For comparison of the techniques, a list of selected advantages and disadvantages for particular methods is summarized in Table 13. Each of the methods involves high-temperature treatment under an inert carbon-containing gas atmosphere. The properties of the formed carbides may be tuned by changing the substrate ratio, time, and temperature of annealing. In the case of the reactive sintering method, the main advantage is the use of the simplest possible substrates that form carbides, e.g., metals, metal oxides, and carbon. However, metals require special storage conditions to prevent oxidation. It is a relatively fast method, where the sintering only lasts up to 10 h; however the required temperatures exceed 1000 °C, and very often 1200 °C. There is a possibility of using lower synthesis temperatures in the case of high-energy milling methods. Due to the high level of reactive surface area development, the annealing after milling may be conducted at temperatures up to 1000 °C. The main disadvantage of the milling technique is the large amount of time consumed for the milling process and the necessity of providing an inert atmosphere in the milling bowl and during the transportation from the bowl to the sintering reactor. The alternative method to high-temperature sintering and high-energy milling is the molten salt method. Due to using the relatively low melting point of the salt acting as the reaction medium, it is possible to carry out the foaming reaction at relatively low temperatures. The disadvantages of this method, however, are that it must be ensured that the salt is properly prepared prior to the reaction. Any moisture and oxygen in the spaces between the salt grains should be properly removed. After synthesis, salt is most often still present, despite being washed several times. The TPR and TPC methods also allow for temperatures lower than those of reactive sintering, but these methods require a carbon-containing gas in a hydrogen atmosphere. Studies with hydrocarbon/hydrogen mixtures require special safety protocols. Despite the high content of carbon impurities, these methods are the most widely used methods for metal carbide preparation. The structure-directing method allows for the preparation of metal carbide particles of a desired morphology using organic precursors. The structure may be directed by the formation of organic–inorganic hybrids such as MoOx-amine, or by deposition of an already formed nanostructure, e.g., nanotubes. This method allows for greater control of the morphology. The main disadvantage of this method is the formation of pyrolysis gases, which are potentially explosive.

**Figure 4:**
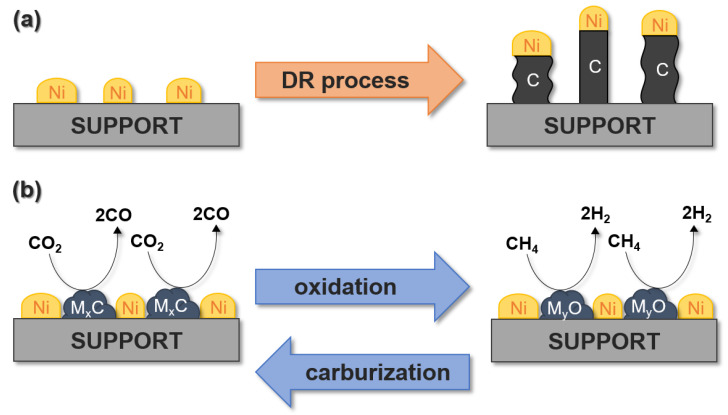
Coke formation on Ni-based catalysts during the DR process (**a**), and the oxidation–recarburization cycle in the case of Ni–M_x_C catalysts (**b**), where M is Mo and W.

Furthermore, TMCs have the advantage that they participate in carburizing–oxidation cycles during the dry reforming process (Figure 4b). During oxidation, CO_2_ is reduced to CO, while during carburization, carbon atoms from methane cracking and CO disproportionation are incorporated into the carbide structure. This prevents the formation of carbon deposits on the catalyst surface. However, it should be noted that carbides such as Mo_2_C and WC show stable activity only at relatively high pressures. At atmospheric pressure, the deactivation of such catalysts occurs in the CO_2_ oxidation process, which is a significant limitation. Therefore, the stability of the catalyst depends on the ability to convert the oxide back to a carbide, which is assisted by high temperatures. Consequently, tungsten and molybdenum carbides can be redox catalysts in dry methane reforming. However, it is worth noting that at atmospheric pressure, the reaction of CO_2_ with such carbides is more favorable than the reaction of CH_4_ with the oxides formed.

Another group of catalysts commercially used in the DRM process is constituted by nickel-based catalysts, which are used because of their lower cost in comparison to noble metals. However, during the decomposition of CH_4_ and disproportionation of CO, these catalysts can be deactivated by forming coke (Figure 4a). The coke formed at the catalyst surface is considered as one of the major factors negatively affecting the catalytic activity of all catalytic processes. However, Sugiyama et al. [225] recently reported the positive effect of coke formation. They investigated Ni/γ-Al_2_O_3_ and Cr_2_O_3_/γ-Al_2_O_3_ catalysts for direct dehydrogenation of isobutane. They found that for the NiO/Al_2_O_3_ catalysts, the formation of coke leads to the formation of nickel carbide coexisting with metallic Ni, which are the main species responsible for the enhanced yield of isobutene formation. Therefore, to avoid the main problems associated with the use of catalysts based only on nickel or TMCs, in recent years, the most commonly used materials are TMCs modified with nickel particles. The addition of a second metal can modify the catalytic performance and structure of carbides. Therefore, core–shell systems are used, that is, carbide cores covered with a metallic coating that prevents oxidation of the carbide surface, thus promoting structural stability. Additionally, Ni-WC is also resistant to oxidation during the process, unlike Ni-Mo_2_C catalysts. However, the combination of molybdenum carbide with other metals such as Ni allows controlled dissociation paths of CO_2_ and CH_4_, ensuring the appropriate conditions for oxidation–recarburization cycles. Titanium carbide is not commonly used as a catalyst in the DR process because it is oxidized to very stable TiO_2_ that does not revert to a carbide form.

Despite the increasing amount of research on the use of TMCs as catalysts in the dry reforming process of hydrocarbons, there are still many areas for improvement. First, most of the research has focused mainly on methane reforming, and only a few studies have been concerned with ethane or other hydrocarbons. Moreover, the research conducted largely lacks data on the characteristics of the catalyst used after the dry reforming process. The catalyst is characterized in detail before it is used in the process, but after its completion, such detailed analyses are not performed. They are mainly limited to the determination of the composition using the XRD method and sometimes to checking changes in the value of the specific surface area. Therefore, in future research on this subject, attention should be paid to this issue. In addition, it is also very important to constantly develop and improve methods of synthesizing TMCs. Currently, an increasing number of new methods are appearing, thanks to which it is possible to obtain TMCs with characteristics favorable for their use in the DR process. However, many of the proposed synthesis techniques require the use of a high temperature, expensive reagents, or specialized equipment, which makes them very difficult to carry out. Therefore, the most interesting alternative to conventional methods of the synthesis of TMCs is the molten salt synthesis technique, which, in the case of carbides, is becoming increasingly popular.

## Figures and Tables

**Table 1:** Important parameters of syngas technologies.

Process	Main Reaction	Enthalpy _Δ_H^0^_298 K_ [kJ/mol]	Pressure [bar]	H_2_/CO Ratio
Dry reforming of methane (DRM)	CH_4_ + CO_2_ = 2CO + 3 H_2_	+247	1	1:1
Steam reforming of methane (STM)	CH_4_ + H_2_O = CO + 3H_2_O	+206	3–25	3:1
Partial oxidation of methane (POM)	CH_4_ + ½ O_2_ = CO + 2H_2_	−35.2	100	2:1
Autothermal reforming (ATR)	CH_4_ + H_2_O = CO + 3H_2_O CH_4_ + ½ O_2_ = CO + 2H_2_	+206 −35.2	1–50	1:1 or 1:2

**Table 2:** Selected process parameters for the synthesis of WC by mechanical milling and the characteristics of obtained particles.

Type of Mill (Balls and Vessel)	Substrates	Ball-to-Powder Weight Ratio	Rotation Speed	Inert Gas Atmosphere	Milling Time	Additional Process Stage	Characteristics of Obtained Particles	Ref.
- planetary mill - WC vessel (250 mL) - WC balls with a diameter of 10 mm	powder mixture (99.9% purity) of W and amorphous C in a stoichiometric amount (6.12 wt.% C)	10:1	300 rpm	Ar	8 h 12 h	-	- crystallites with size of 11 nm (8 h) and 10 nm (12 h)	[62]
- planetary mill - stainless-steel vessel coated with WC-Co - stainless-steel balls coated with WC-Co - vacuum graphite furnace (~10^−4^ atm) for carbothermic reduction	powder mixture of WO_3_ (99.9% purity, ~20 µm) and graphite (99.9% purity, ~1.7 µm)	40:1	250 rpm	-	20 h	(1) Sieving the ground powder through a sieve (125 mesh). (2) Carbothermal reduction (heating rate 10°C/min, reduction temperature 900–1600 °C, cooling rate 20 °C/min).	- lamellar WC	[13]
- hardened steel vessel - hardened steel balls with a diameter of 5–10 mm	powder mixture of WO_3_ (>99% purity), Mg (99.9% purity), and graphite (99.9% purity) at atomic ratio of 1:1:1	20:1 to 50:1	250 rpm	H_2_/Ar	50 h	-	- fine crystalline WC in the form of a powder with a crystallite size in the range of 4–20 nm	[102]
- stainless-steel vessel (80 mL) - 10 steel balls with a diameter of 12 mm	powder mixture (99.9% purity) of W (−75 mesh) and C (−350 mesh)	10:1	n.d.	Ar	71 h 120 h	-	- pure nanocrystalline WC with lenticular-spherical particles, with diameter <100 nm (71 h) and <7 nm (120 h)	[103]
- ball mill with magnetic ball movement control (room temperature, vacuum about 10^−2^ Pa) - 4 hardened stainless-steel balls	powder mixture (8 g) of W (99% purity, mean particle size ~5 µm) and granulated activated carbon (particle size ~0.9–1.7 mm) in the atomic ratio 50:50	30:1	n.d.	-	310 h	(1) Annealing of the ground materials at 1000 °C in a vacuum for 1 h.	- WC in a nanostructured, disordered form - the tendency for WC formation during ball milling was significantly increased after the annealing of the ground materials	[104]

n.d.—no data.

**Table 3:** Selected process parameters for the synthesis of TiC by mechanical milling and the characteristics of obtained particles.

Type of Mill (Balls and Vessel)	Substrates	Ball-to-Powder Weight Ratio	Rotation Speed	Inert Gas Atmosphere	Milling Time	Additional Process Stage	Characteristics of Obtained Particles	Ref.
- water-cooled planetary ball mill - stainless-steel balls with diameter of 5 mm - balls weight: 200 g - stainless-steel vessel with diameter of 50 mm	- Ti powder (99.9% purity, 0.1–1 mm) - graphite (99.9% purity, <20 μm) - Mixture weight: 10 g	20:1	n.d.	Ar	10 min	**Pressureless sintering in a hot press:** The mechanically activated mixture was loaded into a die. The die was set into a hot-pressing plant. P = 1 MPa, T = 900 and 1000°C, t = 30 min, argon atmosphere.	- cubic TiC - aggregates 20–60 μm in size - composition: TiC 87 wt.%, Ti 7.6%, C 5.4% (900 °C), TiC 87.7 wt.%, TiO 6.9%, Ti 2.6%, C 2.8% (1000 °C) - unit cell parameter of TiC: 4.3221 Å (900 °C), 4.3163 Å (1000 °C) - Grain size: 95 nm (900 °C), 81 nm (1000 °C).	[88]
- planetary ball mill - bearing steel vial	- Ti powder (99.9% purity, 74 μm) - graphite (99.9% purity, 250 μm)	40:1	n.d.	Ar	30 h	-	- grain size of 9–11 nm - 2.4% of internal strain - increase in the milling time results in a decrease in the crystallite size	[97]
- high-energy ball mill - three balls with diameter of 20 mm - process pressure: 2.5 atm	- commercial Ti (particle size <50 mesh, 1% impurities: Al 0.32 wt.%, Si 0.37%, V 0.41%) - amorphous carbon black (particle size <250 mesh)	10:1	n.d.	Ar	15, 20 h	-	- TiC particle mean size 25.68 nm (15 h), 20.69 nm (20 h) - increase in the milling time results in a decrease in the crystallite size and in obtaining fine well-distributed powder	[98]
- planetary ball mill - hardened steel balls (4 with diameter of 20 mm and 3 with diameter of 14 mm) - hardened steel vial (150 mL)	- Ti powder (99.5% purity, 5–50 μm) - graphite (99% purity, 5–50 μm) - irregular shape of substrate particles - Ti:C molar ratio of 1:1	10:1	300 rpm	Ar	8, 16 h	Using stearic acid as a PCA (process-control agent) during milling to prevent sticking of the powder to the balls and vial.	- unit cell parameter of TiC: 4.3269 Å (8 h), 4.3139 Å (16 h) - crystallite size: 103.4 nm (8 h), 16.5 nm (16 h), some submicron particles present in the final product - Fe impurities: 0.72 wt.% (8 h), 1.76 wt.% (16 h)	[105]
- magneto ball mill - stainless-steel vial and balls	- Ti powder (99.9% purity, <250 μm) - activated carbon powder (99.9% purity, <150 μm) - Ti:C molar ratio of 50:50, 60:40, and 70:30	n.d.	n.d.	He	60, 82, 96 h	-	- nanocrystalline TiC - sub-stoichiometric composition of TiC - increase in carbon content in substrate mixture results in obtaining TiC with smaller lattice parameter and lower carbon content - unit cell parameter of TiC: 4.321 Å (Ti_50_C_50_, 82 h), 4.310 Å (Ti_60_C_40_, 60 h), 4.302 Å (Ti_70_C_30_, 96 h)	[51]

n.d.—no data.

**Table 4:** Selected process parameters for the synthesis of Mo_2_C by mechanical milling and the characteristics of obtained particles.

Type of Mill (Balls and Vessel)	Substrates	Ball-to-Powder Weight Ratio	Rotation Speed	Inert Gas Atmosphere	Milling Time	Additional Process Stage	Characteristics of Obtained Particles	Ref.
Planetary mill with stainless-steel vials and balls	MoO_3_, Al, graphite at different molar ratios	20:1	300 rpm	Ar	2–150 h	Annealing after the milling under air or Ar atmosphere	- β-Mo_2_C, η-Mo_3_C_2_, and Al_2_O_3_ phases were identified - η-Mo_3_C_2_ transfers into β-Mo_2_C at 700 °C - the content of C in milling mixture determines the carbide crystal phase	[96]
Planetary mill with stainless-steel vials and balls	MoO_3_, graphite	15:1	450 rpm	Ar	5, 10, 15 and 24 h	Pressing at 4–0 MPa, heating, and microwave irradiation (800 W, 60 s). After microwave treatment, annealing at 700 °C and 1000 °C for 30 min	- obtained Mo_2_C particles were contaminated with unreacted MoO_3_ and formed MoO_2_ - the longer the time of milling, the purer the final carbide product	[106]
Planetary mill with stainless-steel vials and balls	MoS_2_, graphite, Na_2_CO_3_	15:1	n.d.	Ar	10, 20, 40, 50, and 70 h	-	- Mo_2_C particles were contaminated with Na_2_S, which was further leached with HCL and hot water	[100]
Planetary ball mill	Graphite, Mo powder, melamine	n.d.	n.d.	n.d.	n.d.	DC arc discharge plasma sintering	- Mo_1.2_C_0.9_ and β-Mo_2_C of orthorhombic structure, graphite phases were observed - nitrogen impurities due to melamine as starting material	[101]
High-energy planetary mill with stainless-steel balls (10 mm)	Carbon black P145 (18–25 nm), (NH_4_)_6_Mo_7_O_24_x 4H_2_O	40:1	600–1000 m/s^2^	yes	30–60 min	Calcination at 760 °C and 800 °C in inert atmosphere	- Mo_2_C phase was obtained surrounded with graphene layers - surface area of product: 125 m^2^/g	[99]

n.d.—no data.

**Table 5:** Selected process parameters for the synthesis of WC by the precursor method and the characteristics of the obtained particles.

Precursors	Preparation of the Precursor Mixture	Carbothermal Reduction				Characteristics of Obtained Particles	Ref.
		Type of Furnace	Flowing Gas	Temperature and Heating Rate	Time		
- ammonium metatungstate (AMT), - glucose	(1) AMT and glucose (>99% purity) were placed in hot distilled water and mixed evenly. (2) The precursor solution was air dried at 30 °C for 36 h. (3) The dried precursor mixture was calcined in a silica tube furnace under argon at 400 °C for 1 h.	vacuum coal tube furnace (3.8 × 10^−2^ Pa)	-	1000 °C 7–10 °C/min	2 h	- single-phase WC nanopowders with a size of 20–80 nm	[61]
- ammonium metatungstate (AMT), - corn starch (MW = 342.29 g/mol)	(1) 5 g of AMT was dissolved in hot deionized water, 4.6 g of corn starch was added and mixed evenly. (2) The mixed solution was added to a Teflon-lined autoclave. (3) The autoclave was sealed in a stainless-steel reactor and kept at 200 °C for 8 h without mixing. (4) The obtained precursor mixture was spray dried with hot air at 250 °C.	vacuum tube furnace	-	980 °C 10 °C/min	1 h	- single-phase WC nanopowders with a diameter of 20–60 nm	[68]
- ammonium metatungstate (AMT), - glucose - CTAB	(1) 4 g of CTAB was dissolved in 20 mL of distilled water and vigorously stirred for 20 min. (2) It was then mixed with 20 mL of an aqueous solution containing glucose and AMT in an AMT:glucose molar ratio of 0.039. (3) The mixture was hydrothermally treated in a Teflon-lined stainless-steel closed autoclave at 180 °C for 24 h to form precursors.	tube furnace	H_2_/N_2_ (V_H2_/V_N2_ 1:3, 50 mL/min)	900 °C	3 h	- *m*-NCTC (mesoporous nano-chain tungsten carbide) with a specific surface of 113 m^2^/g	[129]
- W(CO)_6_ - ethylene glycol (EG) - oleylamine	(1) A mixture of EG (10 mL) and oleylamine (5 mL) was degassed with N_2_ for 5 min at room temperature in a three-necked flask. (2) 250 mg of W(CO)_6_ was added to the mixture of EG and oleylamine and vigorously stirred at 100 °C under N_2_ for 2 h. (3) After cooling, the solution was centrifuged and washed several times with water and ethanol and dried in a vacuum oven at 80 °C for 12 h.	tube furnace	N_2_	800 °C	2 h	- WC with a flake-like structure with an average size of about 250 nm	[130]
- ammonium para- and metatungstate (APT, AMT), - glycine	(1) AMT or AMP and glycine were dissolved in distilled water at 80 °C using magnetic stirrer and obtained solutions were mixed for 1 h. The C/(C+W) relation was equal, 27% (APT) and 28% (AMT). (2) Precursor mixtures were spray dried.	tube furnace	Ar H_2_/Ar (4% vol. H_2_, 96% vol. Ar)	1300 °C 3.3 °C/min	2 h (400 °C) 0.5 h (1300 °C)	- highly agglomerated WC particles - particles size of 10–100 nm	[131]
- H_3_PW_12_O_40_ (PW) - CTAB	(1) 0.5 g of a precursor mixture of 12-phosphotungstic acid (PW) and CTAB was placed in stainless-steel Swagelok cell at room temperature and atmospheric pressure.	tube furnace	-	1000 °C 40 °C/min	10 h	- single-phase WC nanoplatelets and nanorods	[132]
- ammonium paratungstate (APT)/tungsten blue oxide (TBO) - gaseous mixture of CH_4_ and H_2_	(1) TBO was prepared by heating APT after grinding it to reduce particle size at 600 °C for 2 h under N_2_ flow. (2) CH_4_ (99.95%) and H_2_ (99.9995%) were used as the carbon source and reducing agent.	horizontal fixed bed alumina reactor	CH_4_/H_2_ (95% H_2_, 5% CH_4_, 20 l/h)	850 °C (APT) 820 °C (TBO) 5 °C/min	2 h	- highly agglomerated fine WC crystallites - very porous particles (higher porosity in the case of APT) - particle size <1 μm (smaller mean particle size in the case of APT)	[133]
- WO_3_ - iota-carrageenan (IC) - chitin	(1) The dried IC and chitin powders were mixed manually in a weight ratio of 1:4. (2) Nanoparticle WO_3_ was added to the resulting mixture to obtain a WO_3_:C weight ratio of 1:6 and vortexed for 10 min. (3) 5 g of the resulting powder mixture was added to 15 mL of ultrapure water and mixed manually with a spatula. (4) The resulting material resembles a gel and is a precursor to the polymer composite (PCP).	alumina tube furnace	N_2_ (283.17 l/h)	1300 °C 5 °C/min (holding at 300 °C for 30 min to remove excess O_2_)	3 h	- obtained WC was mostly mesoporous, although a small number of macropores were present - the specific surface area was 67.03 m^2^/g - WC grain size was about 20 nm	[134]
- ammonium paratungstate (APT) - multi-walled carbon nanotubes (CNT)	(1) The tungsten was released by wet impregnation of CNT with an aqueous solution (20 mL) of ammonium paratungstate pentahydrate, at a pH close to 5. (2) CNT was added to the aqueous solution of the precursor salt and vigorously stirred at 80 °C for 20 min, before the solvent was slowly evaporated at room temperature. (3) The resulting material was dried overnight at 120 °C and calcined at 350 °C for 2 h.	vacuum furnace (shape memory synthesis)	-	1300 °C 1200 °C	7 h 30 h	- one-dimensional WC without other metals	[135]
- WO_3_ - four types of carbon powders: acetylene black, channel black, activated carbon, graphite	(1) Carbon powder (17.2 %wt.) and WO_3_ powder were ground for 15 h at 180 rpm in a ball mill. (2) WC balls were used to avoid introducing contaminants, and the ball-to-powder ratio was 10:1. (3) Stearic acid was added as a dispersant during milling to prevent agglomeration of the nanoparticles. (4) The resulting WO_3_/C precursor mixture was dried in a vacuum oven.	vacuum furnace (4 Ñ 10^−1^ Pa)	-	1100–1200 °C 10 °C/min	3 h	- acetylene black: - high-purity WC - mean particle size of 100–156 nm - specific surface area of 2.461–3.839 m^2^/g	[136]

**Table 6:** Selected process parameters for the synthesis of molybdenum carbide by the organic–inorganic precursor method and the characteristics of obtained particles.

Substrates for Organic–Inorganic Precursor Preparation	Preparation of the Precursor	Carbothermal Reduction			Characteristics of Obtained Particles	Ref.
		Flowing Gas	Temperature	Time		
- ammonium heptamolybdate - glucose - ammonium carbonate	Ammonium carbonate was added to aqueous solution of ammonium heptamolybdate, followed by addition of glucose. Spray drying.	N_2_	700 °C, 800 °C, and 900 °C	-	- Mo_2_C (crystalline size 5–15 nm) particle-incorporated carbon nanosheets - carbon in form of amorphous phase or stack graphite	[115]
- ammonium heptamolybdate - glucose - hydrochloric acid - hydroquinone	Synthesis of molybdenum blue involving mixing of ammonium. heptamolybdate, hydroquinone or glucose, and hydrochloric acid in different ratios. Drying solution of molybdenum blue particles to xerogel.	N_2_	900 °C	-	- β-Mo_2_C contaminated with η-MoC,C for method with glucose; β-Mo_2_C contaminated with γ-MoC for method with hydroquinone	[114]
- molybdenum chloride - ethanol - urea	Dissolving of MoCl5 in ethanol. Addition of urea. Drying.	N_2_	800 °C	3 h	- depending on urea/molybdenum ratio, diriment crystal phases were obtained: γ-Mo_2_N, α-MoC, and Mo_2_C	[118]
- ammonium molybdate - water - ethanol - ammonia - formaldehyde - tannic acid	Preparation of solution: ammonium heptamolybdate, H_2_O, ethanol, and NH_3(aq)_. Adding formaldehyde dropwise. Addition of tannic acid in water. Aging for 24 h. Centrifugation, drying.	N_2_	900 °C	2 h	- Mo_2_C particles of 5 nm size - Mo_2_C particles were embedded in mesoporous carbon nanosheets	[117]
- ammonium heptamolybdate - Triton X-100 - aniline - pyrrole - ammonium persulfate	Dissolving ammonium heptamolybdate in water-Triton X-100 solution. Addition of aniline and pyrole. Ultrasonication and cooling up to 0 °C in ice bath. Addition of polymerization initiator: (NH_4_)_2_S_2_O_8_. Aging for 12 h. Drying under vacuum at 60 °C.	Ar	650, 750, 850, and 950 °C	3 h	- cubic N-doped Mo_2_C particles coated with amorphous carbon	[121]
- ammonium heptamolybdate - aniline - hydrochloric acid	Dissolving ammonium heptamolybdate in water. Addition of aniline. Setting the pH to pH = 4 with hydrochloric acid. Aging of precipitate at 50 °C for 4 h. Washing, freeze drying.	Inert	600 °C, 700 °C, 800 °C, 900 °C	-	- obtained molybdenum particles exhibited wire-like structure - Mo_2_C and Mo_3_C_2_ phases were determined - carbon shell on Mo_2_C/Mo_3_C_2_ structures	[112]
- natural graphite flake (~150 μm flakes) - ammonium molybdate - poly (propylene glycol) bis(2- aminopropyl ether) (D400)	Synthesis of graphene oxide (GO) from graphite. Preparation of graphene hydrogel by hydrothermal treatment (180 °C, 12 h) of GO and D400 suspension. Impregnation of graphene hydrogel with molybdate salt and hydrothermal treatment (180 °C, 6 h).	n.d.	800°C	2 h	- The final product was N-doped graphene aerogel - Mo_2_C and MoOx identified crystal phases	[137]
- copper acetate - L-glutamic acid - phosphomolybdic acid hydrate - ethanol - 1,3,5-benzenetricarboxylic acid	Preparation of solution containing copper acetate, l-glutamic acid, and phosphomolybdic acid hydrate in water. Addition of 1,3,5-benzenetricarboxylic acid ethanolic solution. Aging for 14 h, washing. Drying at 70 °C. After carburization step, removal of copper particles by washing with iron chloride solution.	N_2_	800°C	6 h	- MoCx particles of nano-octahedron shape - sub-micrometer size of B800 nm	[120]
- ammonium heptamolybdate (AHM) - hexamethylenetetramine (HMT) - γ-Al_2_O_3_ - zeolite Beta - TiO2 - ZrO2	Method I: Preparation of separate AMH and HMT aqueous solutions. Mixing AMH and HMT solutions at 3 °C for 48 h. Separation of precipitate. Method II: Mechanical mixing of precursors prepared according to Method I. Method III: Supported molybdenum carbides: Wetness impregnation of HMT/AHM particles on support from ammonia solution.	N_2_	1st step: 200 °C for 12 h, 2nd step: heating to carburization temperature under inert gas, 3rd step: heating under 20% CH_4_/H_2_	3 h	-	[138]
- ammonium heptamolybdate - aniline - hydrochloric acid	Dissolving ammonium heptamolybdate in water. Addition of aniline. Setting the pH to pH = 4 with hydrochloric acid. Aging of precipitate at 50 °C for 4 h, washing, freeze drying.	Ar	625 °C, 725 °C, 750 °C	5 h	- β-Mo_2_C and α-MoC1-x phases were identified - the transition from α-MoC1-x to β-Mo_2_C with increased carburization temperature - particle size in range of 10–20 nm	[139]

**Table 7:** Selected process parameters for the synthesis of TiC by the MSS method and the characteristics of the obtained particles.

Ti Source	C Source	Salts	Salt:Ti Molar Ratio	Temperature	Time	Characteristic of Obtained Product	Ref.
Ti powder: fine particles (1–3 μm) and large irregular particles (20–40 μm)	- amorphous carbon black (20 nm) - multi-walled carbon nanotubes (8–20 nm) - carbon fiber (uniform diameter of 10 μm) - single-layer graphene resembling crumpled silk veil waves	LiCl-KCl-KF	n.d.	1100 °C (10 °C/min, 5 °C/min from 600 °C)	6 h	- nano-sized single-crystal TiC grains - the type of carbon source does not affect the macroscopic morphology of the obtained TiC	[154]
Ti powder (99% purity)	- amorphous carbon black (N330)	KCl-LiCl	9:1 (salt:reactants mass ratio) C:Ti molar ratio of 2:1	700, 815, 880, 950 °C	2, 3, 4 h	- nanocrystalline TiC at 950 °C for 4 h - at 950 °C, only stoichiometric TiC is formed as a stable phase - increase in heating time results in an increase in purity and crystallinity of the obtained TiC - increase in the temperature results in a decrease in TiC crystallite size - at 950 °C TiC lattice parameter is the closest to the real value	[157]
Ti powder (99.9% purity) with the diameter of 100–150 μm	- Phoenix wood dried and carbonized at 650°C	KCl-KF	2:1, 3:1, 4:1	700–1000 °C	3, 5 h	- TiC coatings on the surface of carbon template - temperature and salt:Ti molar ratio significantly affect the surface and coating density of the TiC coating - whisker-shaped TiC crystals (3:1, 900 °C, 5 h)	[155]
Ti powder (99.8% purity) with the size of 325 mesh	- acetylene black (30–45 nm) - multi-walled carbon nanotubes (diameter of 20–40 nm, length <2 μm, 97% purity) - graphene (average layer 5-6, mean thickness <3 nm, lamellar size of 5–15 μm)	NaCl-KCl	7:1 (salt:reactants mass ratio) 1.5 g of 1:1 Ti:C mixture	750, 800, 850, 900 °C	1, 2, 3, 4 h	- nanocrystalline TiC powder - TiC nanorods and nanosheets - higher temperature and longer synthesis time are more beneficial for TiC formation	[156]
Ti powder (99.5% purity) with the size of 200 mesh	- PAN-based Tenax carbon fiber bundles with the diameter of 6–8 μm	LiCl-KCl-KF	1.5:1, 2.5:1	900 and 950 °C	1–5 h	- high-quality crystalline TiC-coated carbon fibers - homogeneous, thin, and crack-free TiC coating with good flexibility - thickness of about 65 nm - increase in thickness results in stiff and fragile coated fibers - salt components, synthesis time, and salt:Ti molar ratio significantly affect the coating integrity and thickness	[158]

**Table 8:** Selected process parameters for the synthesis of molybdenum carbide by the MSS method and the characteristics of the obtained particles.

Mo and C Source	Salts	Ratio of MS Components	Temperature	Time	Electrochemical Reaction Parameters	Characteristics of Obtained Product	Ref.
Mo and C powders (200 mesh)	NaCl and KCl	equimolar	1000°C	1 h	-	- size of Mo_2_C particles 0.5–1.0 um - Mo metal impurities	[148]
MoS_2_, graphite powder	NaCl and KCl	equimolar	1st step: 800 °C, 2nd step: 700 °C	2 h 10 min	Cell voltage 2.6 V	- nanoparticles of size 30–60 nm - β-Mo_2_C phase deposited on carbon - crystalline size of Mo_2_C 33.9 nm	[159]
Mo foil, CO_2_	CaCl_2_, CaO	CaO:CaCl_2_ = 1:10	850 °C	5 h	Voltage 2.5 V	- MoC and Mo_2_C crystal phases - nanosheet structure	[152]
Mo plate CO_2_	LiCO_3_	-	800 °C	1 h 2 h	Cell voltage 3.1 V	- β-Mo_2_C phase deposited on Mo foil - the height of β-Mo_2_C layer on Mo was 5.8 um after 120 min	[151]
MoO_3_ CFP-carbon fiber paper, carbon black	NaCl, KCl	equimolar	1000 °C	3 h 6 h 9 h	-	- product Ni-doped β-Mo_2_C of flower-like structure - when Ni content was below 10%, Mo_2_C exhibited polyhedral morphology; above 10%, the particle size was reduced and fibrous morphology was observed - the flower-like morphology was crucial for catalytic activity in hydrogen evolution reaction	[150]
Mo powder, carbon nanotubes	LiCl, KCl, KF	mole ratio: 58/40/2	950 °C	1 h		- β-Mo_2_C phase - Mo and graphite impurities	[160]

**Table 9:** Selected synthesis parameters of MAX matrices containing W, Mo, and Ti.

MAX Matrix, Synthesis Method	Substrates	Ratio of Substrates	Preliminary Preparation of Substrates for Synthesis	Temperature and Time of Solid-State Synthesis	Comments	Ref.
Mo_2_Ga_2_C Reactive sintering	Molybdenum and graphite powders, gallium shots	Mo:C molar ratio = 2: 1 Mo_2_C:Ga molar ratio = 1:8	Ball milling of Mo and C powders for 24 h, grinding formed Mo_2_C with gallium	850 °C for 48 h	20% contamination of Mo_2_Ga_2_C phase with unreacted Mo_2_C, Ga, or Ga_2_O_3_	[173]
Mo_3_Al_2_C Reactive sintering	Elemental powders of particular constituents	n.d.	n.d.	24 h at 1500 °C with one intermediate grinding and compacting step, followed by ball milling and hot pressing at 1250 °C and at 56 MPa	Obtained MAX revealed unconventional superconductivity with possibly a nodal structure of the superconducting gap	[183]
Mo_4_ValC_4_ Reactive sintering	Molybdenum (250 mesh), vanadium (325 mesh), vanadium (III) oxide, aluminum (325 mesh), and graphite (325 mesh) powders	Mo:V:V_2_O_3_:Al:C = 4:0.9:0.05:1.2:3.5	Grinding and pestling in agate mortar for 5 min	1650 °C for 4 h under argon atmosphere	The synthesized MAX phase contained impurities of intermetallic and oxide compounds. They were removed by dissolution in 12 M HCl.	[176]
Mo_2_TiAlC_2_ Mo_2_Ti_2_AlC_3_ Reactive sintering	Elemental powders: Mo (325 mesh), Ti (325 mesh), and Al (300 mesh)	mMo:(3-m) Ti:1.1Al:2C, where m = 1.5, 1.8. 2, or 2.2.	Ball milling for 18 h	1600 °C for 4 under Ar flow	The different ratios of starting materials led to formation of different major phases: m ≥ 2 led to (Mo_2_Ti)AlC_2_; for m < 2, (Mo_2_Ti_2_)AlC_3_ was the major product	[172]
(Mo_2/3_Sc_1/3_)_2_AlC Reactive sintering	Elemental powders: graphite, Mo, Al, and Sc	Stoichiometric ratio	Mixing in agate mortar	1500 °C for 20 h under Ar flow	-	[166]
(W,Ti)_4_C_4-x_, x = 1.4 Reactive sintering	Powders of W, Ti, Al, and C	Molar ratio of W:Ti:Al:C = 2:1:1.1:2	n.d.	1600 °C for 4 h	Actual composition ≈ W_2.1(1)_Ti_1.6(1)_C_2.6(1)_	[188]
(W_2/3_Sc_1/3_)_2_AlC (W_2/3_Y_1/3_)_2_AlC Reactive sintering	Elemental powders of W (12 µm), Sc (−200 mesh), Y (40 mesh), Al, and C (−200 mesh)	Stoichiometric ratio	n.d.	1450 °C for 2 h under Ar flow	(W_2/3_Sc_1/3_)_2_AlC sample contained 43 wt.% of (W_2/3_Sc_1/3_)_2_AlC and 31 wt.% of unreacted W	[165]
Ti_2_AlC Hot pressing	TiC (11.8 um), aluminum (11.8 um), active carbon (13.2 um), Ti (10.6 um)	TiC:Ti:Al:C: = 0.5:1.5:1.0:0.5	Mixing in ethanol for 24 h, pressing at high temperatures (1300 °C, 1400 °C, 1450 °C, and 1500 °C) at 30 Mpa under Ar flow	Sintering at pressing temperature: 1300 °C, 1400 °C, 1450 °C, or 1500 °C, the soaking time: 60 min	The main identified phase was Ti_2_AlC; however, with temperature increase (more than 1450 °C), Ti_3_AlC_2_ phase became more significant. Intermetallic impurities of Ti-Al were also detected.	[171]
Ti_3_AlC_2_ Ti_3_SiC_2_ Spark plasma sintering (SPS)	Ti (10.6 um), Si (9.5 um), Al (12.8 um), and TiC (8.4 um)	n.d.	Mixing in ethanol for 24 h	Spark plasma sintering 1150–1300 °C, the soaking time 8 min	High-purity Ti_3_AlC_2_ can be obtained at temperatures 1200–1250 °C and molar ratio of TiC:Ti:Al:Si = 2:1:1:0.2	[170]
Ti_2_AlC, Ti_3_AlC_2_ Molten salt synthesis method	Ti powder (74 um), aluminum (44–420 um), graphite (44 um), sodium chloride, potassium chloride (eutectic phase)	Molar ratio for Ti_2_AlC preparation: Ti:Al:C = 2:1.2:1, for Ti_3_AlC_2_: Ti:Al:C = 3:1.2:2. The salt-to-MAX constituents weight ratio: 1:1	Ball milling (1800 rpm) in heptane to prevent dissolution of salts by adsorbed water fallowed by drying at 95 °C for 8 h and pressing at 140 MPa to form disks	For Ti_2_AlC from 900 to 1000 °C, the reaction time: 2 h; for Ti_3_AlC_2_ 1300 °C, reaction time 2 h	The excess of Al element was required due to its volatility. Formation of MAX phase in shape of globular and long needles.	[189]

n.d.—no data.

**Table 10:** Selected dry methane reforming process parameters and results obtained with the use of tungsten carbide-based catalysts.

Type of Catalyst	Catalyst Properties	Catalyst Synthesis Method	Dry Methane Reforming										Ref.
			Catalyst Mass	Temperature	Pressure	Time	WHSV/ GHSV	CH_4_ /CO_2_ Ratio	CO_2_ Conversion	CH_4_ Conversion	CO Yield	H_2_/CO Ratio	
WC	n.d.	- precursor method (tungsten-promoted biochar) - carbothermal reduction of precursor (3 h, 1000 °C, 50 mL/min of N_2_)	3 g	850 °C	0.5 MPa	500 h	4000–12,000 h^−1^	0.7 0.9 1.0 1.1 1.4	85% 89% 93% 100% 100%	92.5% 88% 82% 78% 76%	83% 90% 92% 86% 79%	0.61 0.69 0.79 0.85 1.03	[199]
α-WC/γAl_2_O_3_	nanorods	- precursor method (nano-WOx supported on γAl_2_O_3_) - carburization of precursor (1 h, 900 °C, 40 mL/min 1/4 *v/v* CH_4_/H_2_)	0.2 g	900 °C	1 atm	5000 TON^1)^	n.d.	1	55%	45%	48%	n.d.	[196]
β-W_2_C/γAl_2_O_3_	nanoparticles								81%	90%	76%	n.d.	
α-WC/W_2_C	39 m^2^/g (CH_4_), 71 m^2^/g (C_2_H_6_)	- temperature-programmed reduction (150 mL/min, 20% vol. CH_4_/H_2_, 877 °C, or 10% vol. C_2_H_6_/H_2_, 627 °C)	n.d.	850 °C	1 bar 8.3 bar	8 h 140 h	2870 h^−1^	1	93.1% 75.4%	92% 62.7%	92.6% 68.6%	0.94 0.79	[210]
WC	20.6 m^2^/g d = 80 nm	- temperature-programmed carburization of precursor WO_3_ (2 h, 800 °C, 50 mL/min, 60/40 *v/v* CH_4_/H_2_)	1.066 g	900 °C 950 °C 970 °C	n.d.	50 h	n.d.	1	61.0% 79.6% 82.8%	28.4% 57.6% 62.2%	n.d.	0.43 0.69 0.71	[211]
WC	d = 18 nm	- plasma-mechanochemical synthesis	1.2 g	843 °C 950 °C	0.867 bar	60 h	n.d.	1	n.d.	34% 55%	n.d.	1.22 1.48	[212]
Co_6_W_6_C	2–3 m^2^/g	- precursor method (Co(en)_3_WO_4_) - reduction in H_2_/Ar and carbidization in flowing CO_2_/CO	0.3 g	850 °C	3.4 atm	100 h	n.d.	1	70%	75%	61%	0.91	[205]
Co_6_W_6_C	5 m^2^/g, particle size < 38 μm	- commercial catalyst	0.3 g	850 °C	5 atm	20 h 90 h	11,200 cm^3^/h/g_cat_	1	78% 78%	82% 81%	76%	1.01 0.99	[213]
Co-βW_2_C/α-WC	438.1 m^2^/g, 0.58 cm^3^/g	- precursor method (activated carbon, AMT) - carburization (2 h, 950 °C, flowing CH_4_/H_2_) - calcination with Co(NO_3_)_2_·6H_2_O (50 mL/min N_2_, 2 h, 600 °C)	2 g	800 °C	1 atm	1 h 10 h	36,000–72,000 cm^3^/h/g_cat_	1	90% 78%	82% 69%	n.d.	0.86 0.69	[214]
Ni-WC	25 m^2^/g, 0.09 cm^3^/g	- precursor method (Ni(NO_3_)_2_·6H_2_O, APT) - carburization of NiW precursor (2 h, 850 °C, 150 mL/min CH_4_/H_2_)	n.d.	800 °C	1 atm	20 h	n.d.	0.67 1.00 1.50	75% 85% 85%	99% 75% 59%	80%^2)^ 83%^2)^ 83%^2)^	0.68 0.79 0.80	[209]
Ni-WC_x_	Ni/W = 0.5 NiW = 4	- precursor method (Ni(NO_3_)_2_·6H_2_O, AMT) - carburization of NiWOx precursor (2 h, 700 °C, 20% vol. CH_4_/H_2_)	0.2 g	800 °C	1 atm		18,000 cm^3^/h/g_cat_	1	71% 68%	58% 55%	n.d.	0.69 0.68	[198]

n.d.—no data; (1) turnover number; (2) H_2_ yield.

**Table 11:** Selected dry methane reforming process parameters and results obtained with the use of molybdenum carbide-based catalysts, CH_4_:CO_2_ = 1.

Type of Catalyst	Mo/C Ratio	Catalyst Synthesis Method	Dry Methane Reforming										Ref.
			Temperature	Pressure	WHSV/ GHSV [mlg^−1^h^−1^]	Sample	CO_2_ Conversion [%]		CH_4_ Conversion [%]		TOS	H_2_/CO Ratio	
							CO_20_	CO_2_	CH_40_	CH_4_			
β-Mo_2_C	0 5 10 15 30 60 100 wt.%	- Mechanical mixing of ammonium molybdate and carbon nanotubes - Heating at 850 °C under Ar	850 °C	atm	18,000	0 5 10 15 30 60 100	90 90 n.d. 90 90 90 5	25 15 n.d. 25 25 25 5	80 85 n.d. 85 85 85 5	15 10 n.d 15. 10 10 5	4 h 5 h n.d. 6 h 14 h 6h 6 h	n.d.	[79]
Mo_2_C	Ascorbic acid/Mo = 1.0	- Reduction of ammonium heptamolybdate with ascorbic acid in acidic medium - Drying of formed molybdenum blue, - calcination at 900 °C under N_2_	650 °C 725 °C 775 °C 825 °C 850 °C	n.d.	30	Mo_2_C	20 30 70 70 75		10 15 35 85 90		n.d.	0.1 0.2 0.35 0.6 0.6	[123]
β-Mo_2_C α-MoC_1-x_	n.d.	- Impregnation of resin with ammonium heptamolybdate by incipient wetness impregnation (IWI) or by ion exchange (IE) or mechanical mixing (MM) - Carbothermal reduction at 900 °C for 1 h under Ar or H_2_ - Passivation with 1% O_2_ in Ar for 2 h	850 °C	atm	6000	IE(Ar) IWI(Ar) MM(Ar) IE(H_2_)	95 95 64 80	95 30 35 25	98 95 55 68	98 25 23 17	12 h 12 h 4 h 4 h	n.d n.d. n.d. n.d.	[78]
Mo_2_C/Al_2_O_3_	5 wt.% Mo, 12.5 wt.% Mo 20 wt.%	- Impregnation of y-Al_2_O_3_ with ammonium heptamolybdate solution at 60 °C - Drying at 120 °C for 24 h - Calcination at 550 °C for 6 h, - TPC under 20%CH_4_/H_2_ flow at 700 °C, 750 °C, or 800 °C	650 °C 750 °C 800 °C	atm	18,000	5Mo 12.5Mo 20Mo	18 20 25		20 25 30		12 h 12 h 12 h	0.6 0.65 0.6	[216]
						5Mo 12.5Mo 20Mo	55 65 60		50 60 60		12 h 12 h 12 h	0.65 0.7 0.72	
						5Mo 12.5Mo 20Mo	85 95 93		85 90 90		12 h 12 h 12 h	0.82 0.85 0.8	
Mo_2_C (me) Mo_2_C (et) Mo_2_C/TiO_2_		- Carburization of MoO_3_ with CH_4_ (me) or C_2_H_6_ (et) in H_2_ - Grinding of MoO_3_ and TiO_2_	850 °C	n.d.	5040	Mo_2_C (me) Mo_2_C (et) Mo_2_C/TiO_2_	99.8 99.9 88.7		92.1 89.5 70.1		n.d.	1.0 1.1 0.9	[229]

**Table 12:** Selected dry methane reforming process parameters and results obtained with the use of nickel-modified molybdenum carbide-based catalysts, CH_4_:CO_2_ = 1.

Type of Catalyst	Mo, Ni, and C Contents/ Ratio	Catalyst Synthesis Method	Dry Methane Reforming										Ref.
			Temperature	Pressure	WHSV/ GHSV [mlg^−1^h^−1^]	Sample	CO_2_ Conversion [%]		CH_4_ Conversion [%]		TOS	H_2_/CO Ratio	
							CO_20_	CO_2_	CH_40_	CH_4_			
Ni-Mo_2_C/MgO	Ni+Mo:β-cyclodextrin = 60:1, Ni: 7 wt.%, Mo: 15 wt.%	- Mixing of Ni and Mo nitrates - Addition of β-cyclodextrin solution - Aging at 80 °C and citric acid addition - Aging at 80 °C, drying at 105 °C, calcination at 550 °C - Passivation with CO_2_	850 °C	1 atm	30,000	Ni-Mo_2_C/MgO	95	95	95	90	200 h	n.d.	[228]
Ni/Mo_2_C/CNT	Ni:Mo = 0.5 (0.5Ni) NI:Mo = 1.0 (1Ni) Ni:Mo = 1.5 (1.5Ni) Ni:Mo = 2.0 (2Ni)	- Impregnation of CNT (carbon nanotubes) with Mo salt - Drying at 70 °C - Calcination at 350 °C in Ar - Impregnation with Ni salt (11.6 wt.%) - Drying at 70 °C, - calcination at 850 °C in Ar	850 °C	1 atm	60,000	0.5Ni 1Ni 1.5Ni 2Ni	85 80 85 85	65 70 25 40	70 70 75 70	65 65 10 30	22 h 36 h 5 h 8 h	n.d.	[222]
Ni-Mo_2_C/La_2_O_3_	Ni: 4.4 wt.% Mo 14.6 wt.%, Ni:Mo = 1:2	- Mixing of ammonium molybdate, nickel nitrate, and lanthanum oxide at 80 °C for 4 h - Drying at 110 °C, - Calcination at 550 °C - Carburization under 20% CH_4_/H_2_ - Passivation in 1% O_2_/Ar for 12 h	800 °C	1 atm	12,000 18,000	Ni-Mo_2_C/La_2_O_3_	70	80	50	60	50 h	n.d.	[224]
Ni-Mo_2_C	Ni:Mo = 0 Ni:Mo = 1 Ni:Mo = 2 Ni:Mo = 3	- Mixing of ammonium molybdate, nickel nitrate, and lanthanum oxide at 80 °C for 4 h - Drying at 110 °C, - Calcination at 550 °C - Carburization under 20%CH_4_/H_2_ - Passivation in 1% O_2_/Ar for 12 h	800 °C	1 atm		Ni:Mo = 0 Ni:Mo = 1 Ni:Mo = 2 Ni:Mo = 3	0 95 92 8	0 70 90 0	0 80 80 40	0 30 80 15	6 h 9 h 22 h	0 0.5 0.52 0.15	[47]
Ni-Mo_2_C/Al_2_O_3_	Al.:urea = 1:2.5 n_Starch_:n_Al. Salt_: 0.125 n_(Ni+Mo)_ = 1.5 Ni content: 15 mol.% Mo content: 0. 3. 5 and 10 mol.%	- Stirring of urea, aluminum nitrate, and starch at 80 °C (solution 1) - Mixing of ammonium molybdate, nickel nitrate, and citric acid (solution 2) - Addition of solution 2 to solution 1 - Stirring for 2.5 h at 80 °C - Drying at 105 °C for 12 h - Calcination under air at 550 °C	800°C	1 at	12,000	0Mo 3Mo 5Mo 10Mo	89 92 92 94	89 93 94 90	84 87 90 85	84 87 88 84	15 h 15 h 15 h 15	0.99 0.97 0.99 0.99	[116]
Mo_2_C-Ni/Al_2_O_3_	Ni:Mo = 2:1 Ni:Mo = 1:2 Ni:Mo = 1:5	- Physical mixing of NiO/Al_2_O_3_ powder with β-Mo_2_C for 60 min	480 °C Plasma treatment	1 atm	50,000	Ni:Mo = 2:1 Ni:Mo = 1:2 Ni:Mo = 1:5	85 7 605	80 75 60	80 75 61	80 75 61	11 h n.d. n.d.	0.9 n.d n.d.	[227]
Ni/MoC_x_/SiO_2_Ni/MoC_x_/Al_2_O_3_Ni/MoC_x_/SiC	20% mol. of Mo, Ni: Mo = 0.2 (0.2NiMo/support) 0.3 (0.3NiMo/support) and 0.4 (0.4NiMo/support)	- Mixing of ammonium molybdate solution with citric acid - Addition of nickel nitrate solution - Impregnation of support using incipient wetness impregnation - Drying at 110 °C for 24 h - Calcination at 550 °C for 4 h - Drying under N_2_ flow at 300 °C - Carburization under 20% CH_4_/H_2_ at 700, 750, and 800 °C	800 °C	1 atm	10,000	0.2NiMo/SiC 0.3NiMo/SiC 0.4NiMo/SiC	90 90 90	85 85 85	85 85 85	75 75 75	0.8 0.8 0.8	20 h	[10]
						0.2NiMo/SiO_2_ 0.3NiMo/SiO_2_ 0.4NiMo/SiO_2_	25 25 75	25 25 90	13 13 65	13 13 75	0.25 0.25 0.75		
						0.2NiMo/Al_2_O_3_ 0.3NiMo/Al_2_O_3_ 0.4NiMo/Al_2_O_3_	25 95 95	12 95 95	12 80 80	5 80 80	0.25 0.8 0.8		

n.d.—no data.

**Table 13:** Advantages and disadvantages of the described methods for TMC preparation.

Method	Advantages	Disadvantages
Reactive sintering	- Appropriate chosen substrate ratio allows for product free from impurities - Relatively fast method	- High-temperature process (above 1000 °C) - Starting materials are mainly pyrophoric metals (Ti, W, Mo) enforcing storage under an inert atmosphere
TPC, TPR	- Lower temperature up to 1000 °C - Fast methods	- Necessity of using explosive hydrocarbon/hydrogen mixture - Carbon impurities
High-energy milling	- High degree of reactive surfaces is due to high level of fragmentation	- Special equipment is necessary - Long time for milling is required - After milling, high-temperature treatment is necessary which is an additional step that must be carried out under an inert atmosphere - Possible impurities from milling balls
Structure-directing method	- Temperature up to 1000 °C - Possibility of formation of micro/nanoparticles of desired shape - Fast method - Developed surface area	- Impurities with excess carbon, - release of dangerous gases during the pyrolysis of the structuring agent
Molten salt synthesis	- Reduction in temperature process in comparison to reactive sintering process	- Impurities with salts - Critical moment of salt degassing
